# The impact of ustekinumab and tumor necrosis factor antagonists on extraintestinal manifestations of Crohn’s disease: a systematic review and meta-analysis

**DOI:** 10.3389/fphar.2026.1849397

**Published:** 2026-07-30

**Authors:** Mao-xuan Lin, Ye-xin Chen, Tu-nan Ding, Wei Han, Mo-han Sun, Yi-yu Dong, Zi-qing Zheng, Yan Zhao, Yi-wen Hu, Run-dong Yu, Yao-fu Zhang, Jun Xu

**Affiliations:** 1 Third Affiliated Hospital, Beijing University of Chinese Medicine, Beijing, China; 2 Beijing University of Chinese Medicine, Beijing, China; 3 Dongzhimen Hospital, Beijing University of Chinese Medicine, Beijing, China; 4 Ningbo Municipal Hospital of Traditional Chinese Medicine (TCM), Affiliated Hospital of Zhejiang Chinese Medical University, Ningbo, Zhejiang, China; 5 Tsinghua University Yuquan Hospital, Beijing, China

**Keywords:** Crohn’s disease (CD), extraintestinal manifestations, meta-analysis, tumor necrosis factor antagonists, ustekinumab

## Abstract

**Objective:**

Extraintestinal manifestations (EIMs) are commonly observed in patients with Crohn’s disease (CD). Although Tumor Necrosis Factor (TNF) antagonists are commonly recommended for the management of several EIMs, their efficacy remains limited. Ustekinumab (UST), an alternative option after TNF antagonists failure, has shown potential efficacy for various EIMs. However, evidence regarding its effectiveness remains inconsistent. This study aimed to evaluate the efficacy and safety of UST and TNF antagonists for CD-related EIMs through a systematic review and meta-analysis.

**Methods:**

PubMed, Embase, and Cochrane Library were searched on 2 August 2025, to identify phase II and III randomized controlled trials (RCTs) evaluating UST or TNF antagonists in adults aged ≥18 years with moderate-to-severe active CD. The primary outcomes were EIMs and EIM-related symptoms. Two investigators, adhering to the PRISMA guidelines, independently screened the literature and extracted data. The risk of bias was assessed using the Cochrane Risk-of-Bias Tool 2.0. Random-effects and fixed-effect models were used to estimate pooled treatment effects. Results were presented using risk ratios (RRs), incidence rate ratios (IRRs) and 95% confidence intervals (CIs).

**Results:**

A total of 23 RCTs, involving 7,810 patients, were included, with EIMs such as fatigue, joint pain, and skin manifestations being considered. The meta-analysis revealed that UST significantly reduced the risk of fatigue in CD patients (IRR 0.53, 95% CI 0.33–0.84, P = 0.008). During maintenance therapy, UST treatment was significantly associated with a reduction in the risk of arthritis/arthralgia (IRR 0.56, 95% CI 0.36–0.86, P = 0.008). Weekly adalimumab (ADA) treatment was significantly associated with an increased risk of fatigue (IRR 2.79, 95% CI 1.12–6.94, P = 0.028).

**Conclusion:**

UST showed potential advantages in the management of fatigue and arthritis/arthralgia. ADA and infliximab (IFX) were not associated with statistically significant benefits for most analyzed EIM outcomes.

**Systematic Review Registration:**

https://www.crd.york.ac.uk/PROSPERO/view/CRD420251111502, identifier CRD420251111502.

## 1 Introduction

Crohn’s disease (CD) is a chronic inflammatory condition of the gastrointestinal tract, characterized by its potential to cause progressive bowel damage and disability (Dolinger et al., 2024). The prevalence of CD is increasing, with approximately one million individuals affected in the United States alone (Lewis et al., 2023). Due to its relapsing systemic inflammatory nature, patients with CD frequently report extraintestinal manifestations (EIMs) (Baumgart and Sandborn, 2012). Compared with patients with ulcerative colitis (UC), those with CD exhibit a higher prevalence of EIMs, ranging from 6% to 47%; these manifestations significantly impact morbidity and quality of life (Harbord et al., 2016; Chams et al., 2019).

In CD, EIMs commonly involve the musculoskeletal system (e.g., peripheral and axial arthritis, enthesitis), skin (e.g., pyoderma gangrenosum (PG), erythema nodosum (EN), Sweet syndrome, aphthous stomatitis), hepatobiliary tract (e.g., primary sclerosing cholangitis (PSC)), and eyes (e.g., episcleritis, anterior uveitis, iritis) (Rogler et al., 2021). However, nearly any system, including hematological, cardiovascular, respiratory, and central nervous systems, can be involved (Gordon et al., 2024a). EIMs are generally considered to occur either in parallel with intestinal disease activity or as independent phenomena unrelated to intestinal inflammation (Trikudanathan et al., 2012). Notably, approximately 25.8% of patients develop EIMs prior to the onset of inflammatory bowel disease (IBD), and the presence of a single EIM is reported to increase the likelihood of developing additional EIMs (Vavricka et al., 2015; Vavricka et al., 2011).

The pathophysiological mechanisms underlying EIMs remain incompletely elucidated. Potential contributing factors may include the extension of immune-mediated responses from the inflamed gastrointestinal tract to other organ systems, alterations in leukocyte trafficking, modifications of the intestinal microbiota, and inherent genetic predisposition (Adams and Eksteen, 2006; Breban et al., 2017; Malik and Aurelio, 2023). A cohort study observed that tumor necrosis factor (TNF) antagonists were generally effective for psoriasis, aphthous stomatitis, uveitis, and peripheral arthritis (Vavricka et al., 2017). For patients with CD and EIMs, TNF antagonists are commonly preferred by gastroenterologists, and the ECCO guidelines recommend the use of TNF antagonists for CD patients with several EIMs (Gordon et al., 2024a). However, prolonged use of TNF antagonists is associated with an increased risk of infections, loss of response, and treatment failure. Exploring diverse biologic therapies for treating EIMs in CD offers patients a wider range of therapeutic options (Min Ho et al., 2019).

Ustekinumab (UST) is a human IgG1κ monoclonal antibody that inhibits the bioactivity of IL-12 and IL-23 by targeting their shared p40 subunit, often serving as an alternative when anti-tumor necrosis factor (anti-TNF) therapy fail (Feagan et al., 2016). Studies have shown that UST effectively induces and maintains clinical remission in patients with moderate-to-severe CD (Almradi et al., 2020). UST has also demonstrated clinical efficacy in the treatment of active psoriatic arthritis (McInnes et al., 2013). A systematic review has indicated that UST is effective, particularly for skin and rheumatologic manifestations (Guillo et al., 2021). However, a *post hoc* analysis showed negative results (Narula et al., 2021). These inconsistent findings highlight the limitations of the current evidence, especially regarding the efficacy of UST for EIMs, for which systematic evaluation and head-to-head comparisons are lacking. Therefore, identifying differences between UST and existing standard treatments in terms of EIMs risk is of critical clinical significance. This study aimed to investigate the impact of UST and TNF antagonists on the risk of EIMs in CD. Through a systematic review and meta-analysis of published randomized controlled trials, we sought to provide a more comprehensive and reliable evidence base for positioning UST in the management of EIMs.

## 2 Methods

This systematic review was performed according to an a priori established protocol and guidance from the Cochrane Handbook, and reported according to the Preferred Reporting Items for Systematic Reviews and Meta-Analyses (PRISMA) statement guidelines (Higgins et al., 2024a; Moher et al., 2009). The meta-analysis was registered with the International Prospective Register of Systematic Reviews (PROSPERO; CRD420251111502). The data that support the findings of this study are available from the corresponding author upon reasonable request.

### 2.1 Data sources and search strategy

PubMed, Embase, and Cochrane Library were systematically searched for articles published from inception to 2 August 2025, using the MeSH terms summarized in Supplementary Table S1. Results were limited to the English language. No date limits were applied. The reference list of each included paper and relevant review articles were also reviewed to ensure completeness of the search. Two reviewers (R.D. and Y.Y.) independently screened all records in duplicate to determine study eligibility. Studies were initially screened by title and abstract using pre-established screening questions. To ascertain whether EIMs or EIM-related outcomes were reported, the full text, Supplementary Appendix, and ClinicalTrials.gov record were reviewed for each randomized controlled trial (RCT) study that met inclusion criteria related to the population, intervention, and comparator. Disagreements between two researchers were resolved through discussion with a third reviewer (M.X.) (Haddaway et al., 2022).

### 2.2 Eligibility criteria

Studies were considered eligible if they (1) were RCTs; (2) included adults older than 18 years; (3) evaluated UST or anti-TNF therapy based on guideline recommendations and findings from phase II/III RCTs compared with controls; (4) reported EIMs (haematological, cardiovascular, respiratory disease; hepatobiliary manifestations; bone and joint disease; skin disease; ocular manifestations; central nervous system manifestations; fatigue; endocrine manifestations (Gordon et al., 2024b)); Certain symptoms, such as headache, were extracted and analyzed as prespecified clinically relevant EIM-related symptoms. Eligible TNF antagonists included the following agents: adalimumab (ADA) and infliximab (IFX). These agents are strongly recommended as induction therapy for moderate-to-severe active CD and also used to treat dermatologic manifestations such as psoriasis, PG, and EN, and rheumatologic manifestations such as arthralgias and psoriatic arthritis (Gordon et al., 2024a; Gordon et al., 2024b; Ellis and Azmat, 2023; Aboobacker et al., 2025). Controls were defined as placebo. Trials evaluating the effect of UST and TNF antagonists in a population with a prior diagnosis of EIMs in CD were excluded.

### 2.3 Data extraction

Data were extracted in duplicate by two independent reviewers (R.D. and H.W.) using a standardized spreadsheet (Excel, Microsoft). For each study, we abstracted the title, year of publication, drug (class, drug name, and dose), control, design (open label or placebo controlled), intervention and control numbers, outcomes, and risk of bias. It would be obtained by extrapolation or by trying to communicate with the original author if any data was missing (Li et al., 2024). Outcomes were reported from the point of longest available follow-up. Discrepancies encountered during screening or data extraction were resolved by discussion and consensus, or recourse to a third reviewer (M.X.).

### 2.4 Outcomes

The primary outcomes of this meta-analysis were EIMs and EIM-related symptoms during follow-up. Outcomes reported in individual studies were grouped into “domains” according to underlying pathomechanisms and affected organ systems; we also analysed individual designations where possible. All reported types were considered. Secondary outcomes included adverse events and serious adverse events.

### 2.5 Risk-of-bias assessment

A quality assessment was performed using version two of the Cochrane risk-of-bias tool for randomized trials (Higgins et al., 2024b). For the possible sources of bias risk arising from improper experimental methods or the limitations of the sample itself in the research process, three evaluations are given: high risk, some concerns, and low risk. If one of the domains was rated as high risk, the study was considered at a high risk of bias. Two independent reviewers (Z.Q. and Y.Y.) performed risk-of-bias assessments, and disagreements were resolved by a third reviewer (Y.X.).

### 2.6 Data synthesis and analysis

In studies with multiple dosage groups, outcome data from the intervention groups were combined. All outcomes of interest were dichotomous. When person-years data were available, incidence rate ratios (IRRs) or risk ratios (RRs) with 95% confidence intervals (CIs) were used. If event rates were low (<10%) and sample sizes large, the RR was approximated by the IRR (Higgins and Thompson, 2002; Fleiss, 1993). For event rates slightly above 10%, a sensitivity analysis excluding such studies was performed. Heterogeneity between studies was assessed using the I^2^ statistic, τ^2^ value, and Q statistic. A fixed-effect model was used to summarize studies when I^2^ < 40%, while a random-effects model was employed when I^2^ ≧ 40%. The results of the meta-analysis were visualized using forest plots. Regardless of statistical heterogeneity, pre-specified subgroup analyses by drug dosage and induction/maintenance therapy were conducted when evidence was available. Funnel plots were planned to assess publication bias if 10 or more studies were included, but this condition was not met. All statistical analyses were performed using R (R Core Team 2024, v4.3.3), using the “meta” package for meta-analysis and influence analysis (Balduzzi et al., 2019).

### 2.7 Assessment of the credibility of evidence

The GRADE (Grading of Recommendations, Assessment, Development, and Evaluation) method was used to assess the quality of evidence for both primary and secondary outcomes (Guyatt et al., 2008). Initially, the results of RCTs was considered high-quality evidence. However, the quality could be downgraded due to factors such as risk of bias, indirectness of evidence, unexplained heterogeneity, publication bias, or sparse data. For each comparison, two team members independently rated the quality of evidence for each outcome (i.e., the estimated effect) as high, moderate, low, or very low. Disagreements were resolved through consensus, and if necessary, arbitration by a third team member was used to settle differences.

## 3 Results

A total of 37,971 records were identified through literature searches and other sources. After removing duplicates, records were screened by title and abstract, and 25,176 were excluded. A full-text and ClinicalTrials.gov review was conducted for 592 articles, resulting in the inclusion of 23 studies (Feagan et al., 2016; Colombel et al., 2007; Rutgeerts et al., 2012; Sandborn et al., 2007a; Chen et al., 2020; Watanabe et al., 2014; Sandborn et al., 2007b; Watanabe et al., 2012; Hanauer et al., 2006; Sandborn et al., 2008; Sandborn et al., 2012; Sandborn et al., 2022; Ferrante et al., 2024; Hanauer et al., 2024; Regueiro et al., 2016; Present et al., 1999; Sands et al., 2004; Rutgeerts et al., 1999; Regueiro et al., 2009; Hanauer et al., 2002; Colombel et al., 2010; Targan et al., 1997) (reported in 235 publications), involving 7,810 adults with moderate-to-severe active CD in the qualitative and quantitative synthesis. The study selection process is detailed in Supplementary Figure S1.

### 3.1 Study characteristics

The 23 eligible RCTs were published between 1999 and 2022. One trial (Chen et al., 2020) was conducted in China, one (Regueiro et al., 2009) in the United States, two (Watanabe et al., 2014; Watanabe et al., 2012) in Japan, and 19 were international studies. All trials employed placebo and assessed the efficacy and safety of UST (n = 6) and TNF antagonists (n = 17; ADA, IFX). The number of participants in the included studies ranged from 24 to 1,065. All studies reported adverse events, from which EIMs and EIM-related symptoms were extracted where available. Two studies (Sands et al., 2004; Regueiro et al., 2009) reported all adverse events, four studies (Rutgeerts et al., 2012; Present et al., 1999; Rutgeerts et al., 1999; Targan et al., 1997) reported adverse events occurring in ≥5% of patients in any group, and 17 studies reported adverse events occurring in ≥10% of patients, mainly including fatigue, headache, arthritis/arthralgia, anemia, and skin and ocular manifestations (Supplementary Table S2). Baseline characteristics of the included studies are shown in Table 1.

**TABLE 1:** Baseline characteristics of included studies.

Table characteristics of included trials													
Source	Trial design	Study population	Country	No.of participants	Intervention			Patient characteristics				Follow-up, PYs	Primary outcome definition
								Mean age, y^a^	Activity score^a^ ^,^ ^b^	CRP (mg/dL)	No. of female participants (%)		
Adalimumab													
Colombel et al. (2007) CHARM	Double-blind, randomized, placebo-controlled RCT	Moderately to severely active CD	Multicenter, global (92 sites)	854	Placebo (n = 261)			37.1 (11.9)	313.1 (62.0)	2.3 (3.4)	528 (61.8)	697.48	Reported asadverse event
					Adalimumab 40 mg every other week (n = 260)								
					Adalimumab 40 mg weekly (n = 257)								
Rutgeerts et al. (2012) EXTEND	Quadruple-blind, randomized, placebo-controlled RCT	Moderately to severely active CD	Multicenter, global (19 sites)	129	Placebo (n = 65)			37.2 (12.60)	321.1 (72.1)	Not reported	41 (63.1)	55.60	Reported asadverse event
					Adalimumab 40 mg every other week (n = 64)			37.1 (11.10)	318.7 (68.6)		40 (62.5)		
Sandborn et al. (2007a) CLASSIC II	Quadruple-blind, randomized, placebo-controlled RCT	Moderately to severely active CD	Multicenter, global (53 sites)	55	Placebo (n = 18)			36 (13)	107 (62)	0.2 (0.2)	12 (67)	59.24	Reported asadverse event
					Adalimumab 40 mg every other week (n = 19)			34 (12)	106 (33)	0.8 (0.8)	12 (63)		
					Adalimumab 40 mg weekly (n = 18)			38 (10)	88 (50)	0.6 (0.9)	9 (50)		
Chen et al. (2020)	Quadruple-blind, randomized, placebo-controlled RCT	Moderately to severely active CD	China (15 sites)	205	Placebo (n = 103)			32.6 (9.5)	274.7 (49.1)	2.71 (3.15)	30 (29.1)	15.76	Reported asadverse event
					Adalimumab 160/80 mg (n = 102)			33.2 (10.2)	272.1 (48.1)	2.39 (2.46)	35 (34.3)		
Watanabe et al. (2014)	Quadruple-blind, randomized, placebo-controlled RCT	Moderately to severely active CD	Japanese (29 sites)	50	Placebo (n = 25)			30.80 (10.939)	Not reported	Not reported	10 (40.0)	29.00	Reported asadverse event
					Adalimumab 40 mg every other week (n = 25)			31.60 (7.171)			9 (36.0)		
Sandborn et al. (2007b)	Double-blind, randomized, placebo-controlled RCT	Moderately to severely active CD, failed treatment/intolerance to adalimumab	Multicenter, global (52 sites)	325	Placebo (n = 166)			37 (12)	313 (66)	Not reported	101 (60.8)	24.99	Reported asadverse event
					Adalimumab 160/80 mg (n = 159)			39 (12)	313 (58)		109 (68.6)		
Watanabe et al. (2012)	Quadruple-blind, randomized, placebo-controlled RCT	Moderately to severely active CD, or failed treatment/intolerance to adalimumab	Japanese (18 sites)	90	Placebo (n = 23)			30.4 (6.93)	Not reported	Not reported	7 (30.4)	6.92	Reported asadverse event
					Adalimumab 80 mg/40 mg (n = 34)			30.6 (9.26)			18 (52.9)		
					Adalimumab 160 mg/80 mg (n = 33)			32.0 (9.60)			13 (39.4)		
Hanauer et al. (2006) CLASSIC-I	Double-blind, randomized, placebo-controlled RCT	Moderately to severely active CD	Multicenter, global (55 sites)	299	Placebo (n = 74)			37 (13)	296 (60)	1.8 (2.6)	37 (50.0)	22.99	Reported asadverse event
					Adalimumab 80 mg/40 mg (n = 75)			38 (12)	301 (61)	2.0 (2.8)	50 (66.7)		
					Adalimumab 160 mg/80 mg (n = 76)			39 (11)	295 (52)	1.4 (1.9)	40 (52.6)		
					Adalimumab 40 mg/20 mg (n = 74)			39 (13)	299 (57)	1.6 (2.1)	35 (47.2)		
Ustekinumab													
Feagan et al. (2016) UNITI-1	Quadruple-blind, randomized, placebo-controlled RCT	Moderately to severely active CD, inadequate response/intolerance to tumor necrosis factor antagonists	Multicenter, global (177 sites)	769	Placebo (n = 256)			37.6 (11.86)	Not reported	Not reported	135 (52.7)	118.15	Reported asadverse event
					Ustekinumab 130 mg (n = 254)			37.4 (11.74)			153 (60.2)		
					Ustekinumab approximately (∼) 6 (mg/kg) (n = 259)			37.2 (12.57)			152 (58.7)		
Feagan et al. (2016) UNITI-2	Quadruple-blind, randomized, placebo-controlled RCT	Moderately to severely active CD, inadequate response/intolerance to conventional therapy	Multicenter, global (224 sites)	640	Placebo (n = 214)			40.1 (13.09)	Not reported	Not reported	113 (52.8)	98.30	Reported asadverse event
					Ustekinumab 130 mg (n = 213)			39.2 (13.74)			122 (57.3)		
					Ustekinumab Approximately (∼) 6 (mg/kg) (n = 213)			38.8 (13.65)			342 (53.4)		
Sandborn et al. (2008) C0379T07	Triple-blind, randomized, placebo-controlled RCT	Moderately to severely active CD, failed treatment/intolerance to adalimumab	Multicenter, global (42 sites)	104	Placebo (n = 26)			37 (14)	292 (40)	1.2 (1.4)	11 (42.3)	16.00	Reported asadverse event
					Subcutaneousustekinumab 90 mg (n = 25)			37 (13)	311 (80)	1.7 (2.3)	10 (40.0)		
					Placebo (n = 27)			44 (11)	316 (56)	1.3 (1.3)	14 (51.9)		
					Intravenous ustekinumab 4.5 mg/kg (n = 26)			43 (12)	325 (66)	1.3 (2.1)	12 (46.2)		
Sandborn et al. (2012) CERTIFI	Double-blind, randomized, placebo-controlled RCT	Moderately to severely active CD, inadequate response/intolerance to tumor necrosis factor antagonists	Multicenter, global (153 sites)	526	Placebo (n = 132)			39.5 (13.1)	312.4 (64.2)	Not reported	68 (51.5)	260.60	Reported asadverse event
					Ustekinumab 1 mg/kg (n = 131)			38.8 (12.0)	318.5 (62.4)		83 (63.4)		
​	​	​	​	​	Ustekinumab 3 mg/kg (n = 132)			38.2 (12.6)	326.8 (63.1)	​	75 (56.8)	​	​
					Ustekinumab 6 mg/kg (n = 131)			39.4 (13.2)	338.0 (67.3)		83 (63.4)		
Sandborn et al. (2022) IM-UNITI	Quadruple-blind, randomized, placebo-controlled RCT	Moderately to severely active CD, inadequate response/intolerance to conventional therapy	Multicenter, global (220 sites)	397	Placebo (n = 133)			39.5 (12.69)	135.0^c^	0.428^c^	74 (55.6)	1965.95	Reported asadverse event
					Ustekinumab 90 mg every 12 weeks (n = 132)			38.6 (13.65)	134.0^c^	0.516^c^	74 (56.1)		
					Ustekinumab 90 mg every 8 weeks (n = 132)			37.9 (13.2)	127.0^c^	0.446^c^	76 (57.6)		
Ferrante et al. (2024) VIVID-1	Triple-blind, randomized, placebo-controlled RCT	Moderately to severely active CD, Moderately to severely active CD, inadequate response/intolerance to conventional therapy	Multicenter, global (324 sites)	1065	Placebo (n = 199)			36.3 (12.7)	318.9 (86.2)	0.76 (0.29–1.88)^d^	81 (40.7)	412.80	Reported asadverse event
					Mirikizumab 900 mg/300 mg (n = 579)			36.0 (13.2)	323.1 (85.8)	0.85 (0.29–2.50)^d^	247 (42.7)		
					Ustekinumab 6 mg/kg, subcutaneous ustekinumab 90 mg every 8 weeks (n = 287)			36.6 (12.7)	318.5 (93.2)	0.89 (0.34–2.48)^d^	150 (52.3)		
Infliximab													
Hanauer et al. (2024) LIBERTY-CD	Quadruple-blind, randomized, placebo-controlled RCT	Moderately to severely active CD, inadequate response or intolerance to immunosuppressants or corticosteroids	Multicenter, global (114 sites)	343	Placebo (n = 112)			32.3 (11.53)	309.69 (62.615)	2.129 (3.298)	43 (38.4)	289.40	Reported asadverse event
					Subcutaneous infliximab 120 mg (n = 231)			36.0 (12.53)	311.95 (58.433)	1.889 (3.695)	97 (42.0)		
Regueiro et al. (2016)	Quadruple-blind, randomized, placebo-controlled RCT	Moderately to severely active CD	Multicenter, global (104 sites)	297	Placebo (n = 150)			35.4 (12.41)	109.8 (54.75)	Not reported	69 (46.0)	509.09	Reported asadverse event
					Infliximab 5 mg/kg (n = 147)			37.1 (13.49)	107.7 (52.75)		70 (47.6)		
Present et al. (1999)	Double-blind, randomized, placebo-controlled RCT	Moderately to severely active CD	Multicenter, global (12 sites)	94	Placebo (n = 31)			35.4 (8.6)	192.9 (92.0)	Not reported	14 (45.2)	32.54	Reported asadverse event
					Infliximab 5 mg/kg (n = 31)			41.2 (12.2)	184.4 (98.5)		36 (57.1)		
					Infliximab 10 mg/kg (n = 32)			35.0 (12.3)	184.9 (97.5)				
Sands et al. (2004)	Double-blind, randomized, placebo-controlled RCT	Moderately to severely active CD, single or multiple draining fistulas	Multicenter, global (45 sites)	282	Infliximab 5 mg/kg (n = 282)	Had a response (n = 195)	Placebo (n = 99)	36 (29–46)^d^	Not reported	0.7 (0.4–2.2)^d^	51 (51.5)^d^	216.92	Reported asadverse event
							Infliximab 5 mg/kg (n = 96)	37 (28–47)^d^		0.6 (0.4–1.5)^d^	43 (44.8)^d^		
						Had no response (n = 87)	Placebo (n = 44)	40 (31–48)^d^		0.9 (0.5–2.1)^d^	44 (50.6)^d^		
							Infliximab 5 mg/kg (n = 43)						
Rutgeerts et al. (1999)	Double-blind, randomized, placebo-controlled RCT	Moderately to severely active CD	Multicenter, global (17 sites)	73	Placebo (n = 36)			39^d^	Not reported	Not reported	13 (36.1)	43.32	Reported asadverse event
					Infliximab 10 mg/kg (n = 37)			34^d^			22 (59.5)		
Regueiro et al. (2009)	Double-blind, randomized, placebo-controlled RCT	Moderately to severely active CD, curative resection/ileocolonic anastomosis for CD	America (1 site)	24	Placebo (n = 13)			32^d^	112^d^	0.1^d^	3 (23.1)	24.00	Reported asadverse event
					Infliximab 5 mg/kg (n = 11)			43^d^	202^d^	0.5^d^	5 (45.5)		
Hanauer et al. (2002) ACCENT I	Double-blind, randomized, placebo-controlled RCT	Moderately to severely active CD	Multicenter, global (55 sites)	573	Placebo (n = 188)			35 (28–46)^e^	297 (260–342)^e^	0.8 (0.4–2.3)^e^	334 (58)	543.30	Reported asadverse event
					Infliximab 5 mg/kg (n = 193)								
					Infliximab 5 mg/kg, 10 mg/kg every 8 weeks (n = 192)								
Colombel et al. (2010) SONIC	Triple-blind, randomized, placebo-controlled RCT	Moderately to severely active CD, inadequate response or intolerance to corticosteroids	Multicenter, global (92 sites)	508	Azathioprine + Placebo (n = 170)			36.3 (12.92)	287.2 (52.9)	1.0^f^	80 (47.1)	250.30	Reported asadverse event
					Infliximab + Placebo (n = 169)			36.6 (13)	284.8 (62.1)	1.1^f^	85 (50.3)		
					Infliximab + Azathioprine (n = 169)			35.9 (11.97)	289.9 (55.0)	1.0^f^	81 (47.9)		
Targan et al. (1997)	Double-blind, randomized, placebo-controlled RCT	Moderately to severely active CD	Multicenter, global (18 sites)	108	Placebo (n = 25)			38.5 (11.0)	288 (54)	1.28 (1.39)	10 (40.0)	30.55	Reported asadverse event
					cA2 5 mg/kg (n = 27)			37.0 (11.8)	312 (56)	2.21 (2.36)	13 (48.1)		
					cA2 10 mg/kg (n = 28)			39.3 (10.6)	318 (59)	2.32 (3.42)	15 (53.6)		
					cA2 20 mg/kg (n = 28)			36.0 (9.7)	307 (50)	2.24 (2.39)	15 (53.6)		

CD, Crohn’s disease, CRP C-reactive protein, SD, standard deviation; RCT, randomized clinical trial; IQR, interquartile range; PYs, patient years.

^a^ Data are presented as mean (SD) unless otherwise specified.

^b^ Crohn’s disease disease activity index for Crohn’s disease or Mayo score for ulcerative colitis.

^c^ Data are presented as median.

^d^ Data are presented as median (IQR).

^e^ Data are presented as median (range).

^f^ Data are presented as mean.

### 3.2 Risk of bias

The Cochrane risk of bias assessments for the RCTs are presented in Supplementary Figures S2 and S3. Among the included studies, 13 studies were rated as low risk of bias, 10 studies were rated as having some concerns, and no studies were rated as high risk of bias. Concerns related to outcome measurement were identified in all studies. Because EIMs were not clearly defined and the methods used to derive the reported maximum event counts were unclear. The number of studies for each comparison was small, and we could not reliably detect publication bias.

### 3.3 Results of syntheses

#### 3.3.1 Fatigue

**FIGURE 1:**
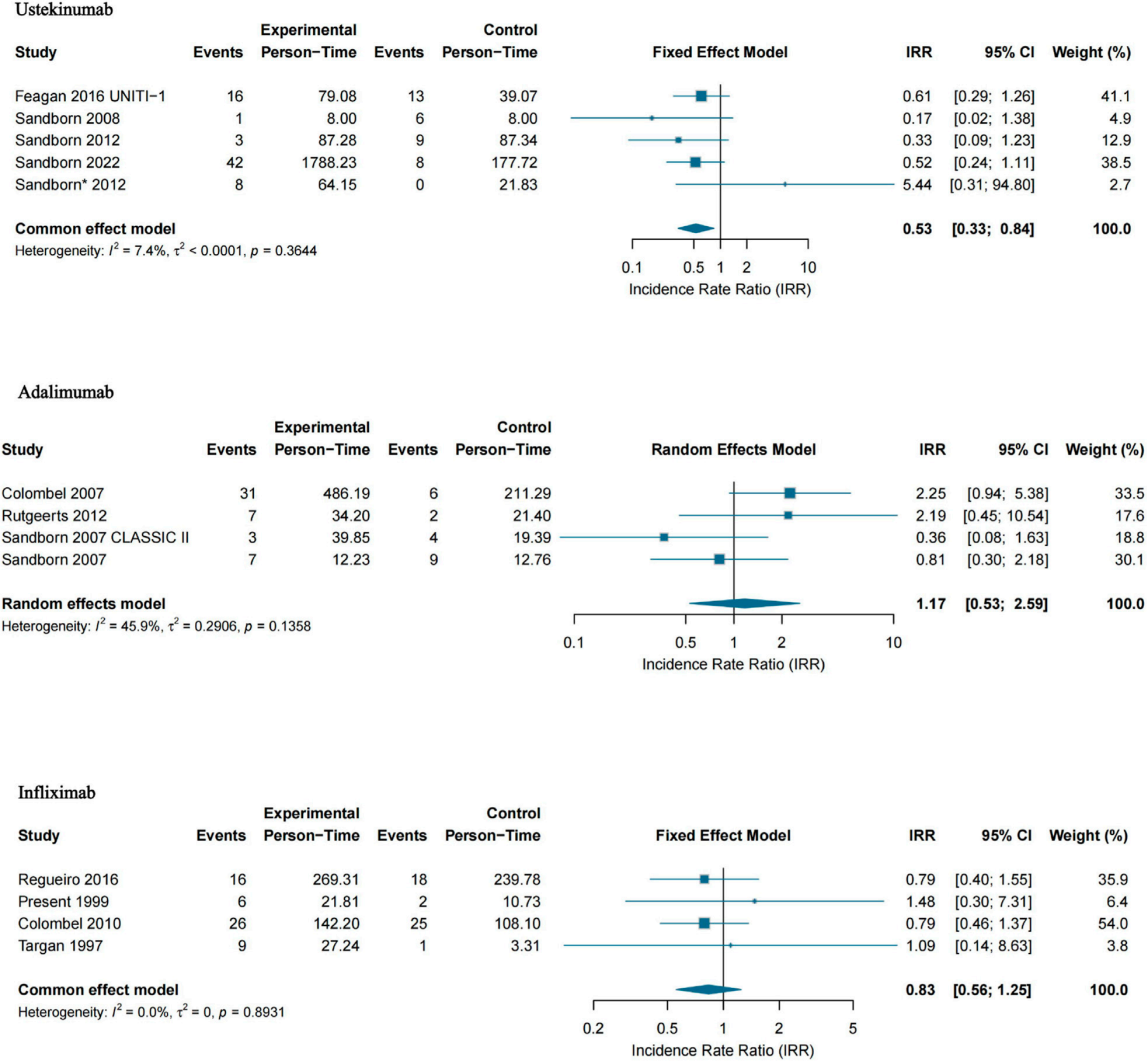
Forest plot illustrating IRR of ustekinumab, adalimumab and infliximab on fatigue vs. placebo. CI confidence interval.

Thirteen trials reported fatigue as an adverse event during follow-up. UST treatment was significantly associated with a reduced risk of fatigue (IRR 0.53, 95% CI 0.33–0.84, P = 0.008, moderate quality evidence; Figure 1; Supplementary Table S3). ADA treatment was associated with an increased risk of fatigue, although not statistically significant (IRR 1.17, 95% CI 0.53–2.59, very low certainty evidence; Figure 1; Supplementary Table S3). No statistically significant difference was found between IFX and placebo for the risk of fatigue (IRR 0.83, 95% CI 0.56–1.25, low certainty evidence; Figure 1; Supplementary Table S3).

#### 3.3.2 Headache

**FIGURE 2:**
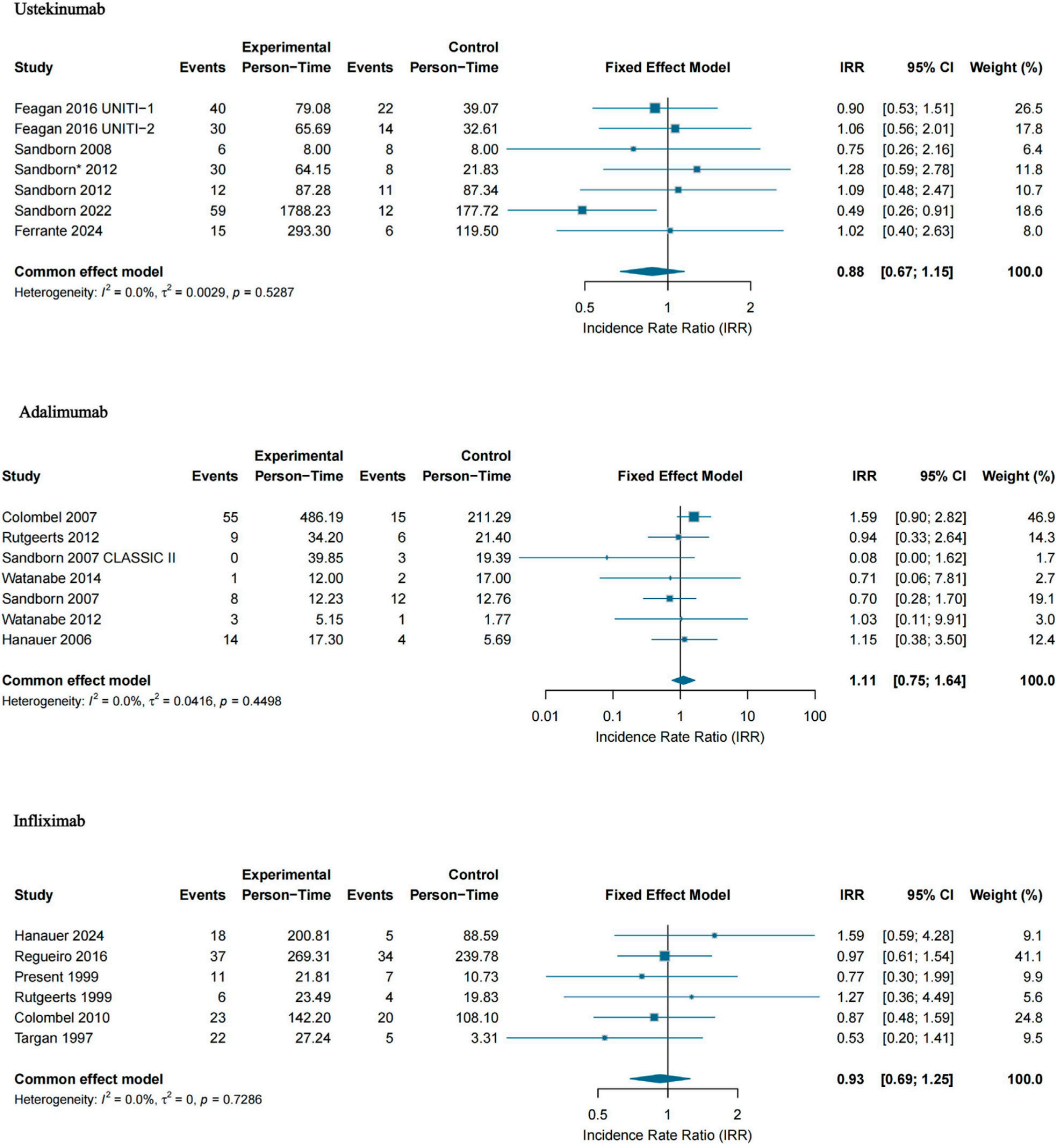
Forest plot illustrating IRR of ustekinumab, adalimumab and infliximab on headache vs. placebo. CI confidence interval.

Twenty trials reported headache as an adverse event during follow-up. No statistically significant difference was found between UST and placebo in the risk of headache (IRR 0.88, 95% CI 0.67–1.15, moderate certainty evidence; Figure 2; Supplementary Table S4). Neither ADA and IFX was associated with a statistically significant difference in the risk of headache compared with placebo (ADA IRR 1.11, 95% CI 0.75–1.64, low certainty evidence; IFX IRR 0.93, 95% CI 0.69–1.25, moderate certainty evidence; Figure 2; Supplementary Table S4).

#### 3.3.3 Arthritis/arthralgia

**FIGURE 3:**
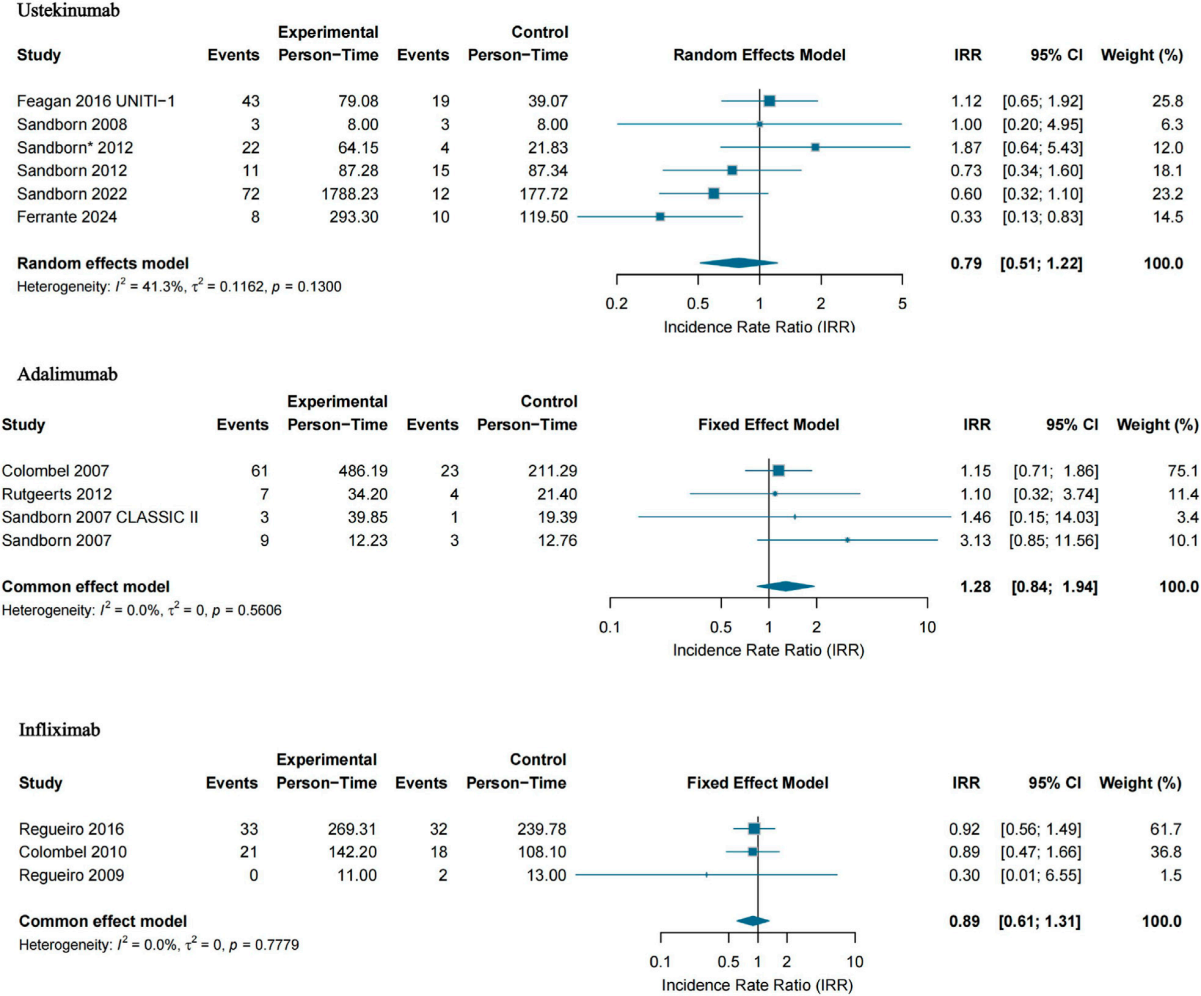
Forest plot illustrating IRR of ustekinumab, adalimumab and infliximab on arthritis/arthralgia vs. placebo. CI confidence interval.

Thirteen trials reported arthritis or arthralgia as an adverse event during follow-up. No statistically significant difference in the risk of arthritis/arthralgia was observed between UST and placebo (IRR 0.79, 95% CI 0.51–1.22, moderate certainty evidence; Figure 3; Supplementary Table S5). ADA treatment was associated with an increased risk of arthritis/arthralgia, though not statistically significant (IRR 1.28, 95% CI 0.84–1.94, very low certainty evidence; Figure 3; Supplementary Table S5). No significant difference in the risk of arthritis/arthralgia was found between IFX and placebo (IRR 0.89, 95% CI 0.61–1.31, low certainty evidence; Figure 3; Supplementary Table S5).

#### 3.3.4 Anemia

**FIGURE 4:**
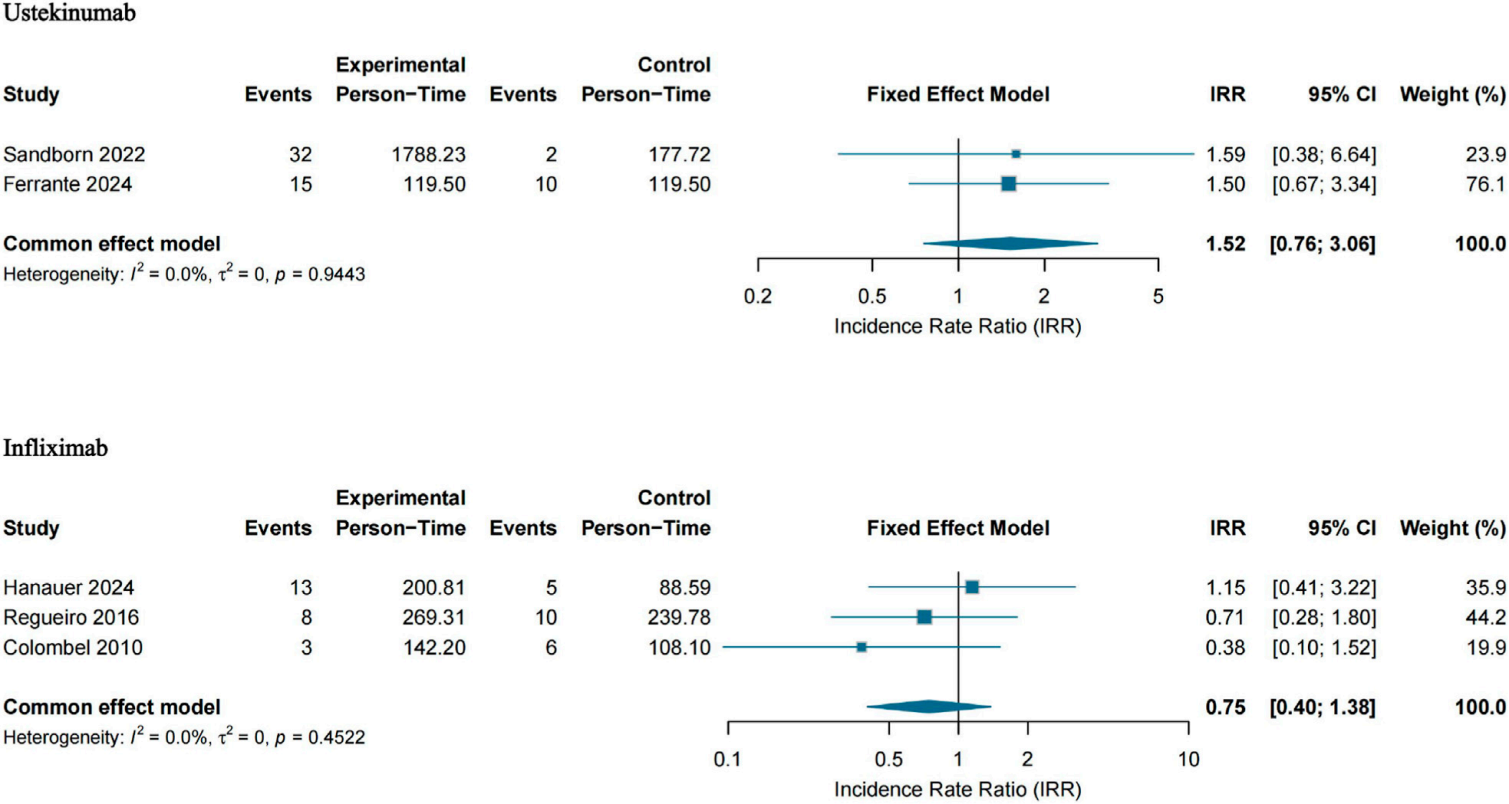
Forest plot illustrating IRR of ustekinumab and infliximab on anaemia vs. placebo. CI confidence interval.

Two RCTs (IM-UNITI, VIVID-1) (Sandborn et al., 2022; Ferrante et al., 2024) reported anemia as an adverse event in patients treated with UST. In the pooled analysis, no statistically significant difference in the risk of anemia was found between UST and placebo (IRR 1.52, 95% CI 0.76–3.06, low certainty evidence; Figure 4; Supplementary Table S6). Three studies (Hanauer et al., 2024; Regueiro et al., 2016; Colombel et al., 2010) reported anemia in patients treated with IFX, with no statistically significant difference in the risk of anemia compared to placebo (IRR 0.75, 95% CI 0.40–1.38, low certainty evidence; Figure 4; Supplementary Table S6).

#### 3.3.5 Skin manifestations

**FIGURE 5:**
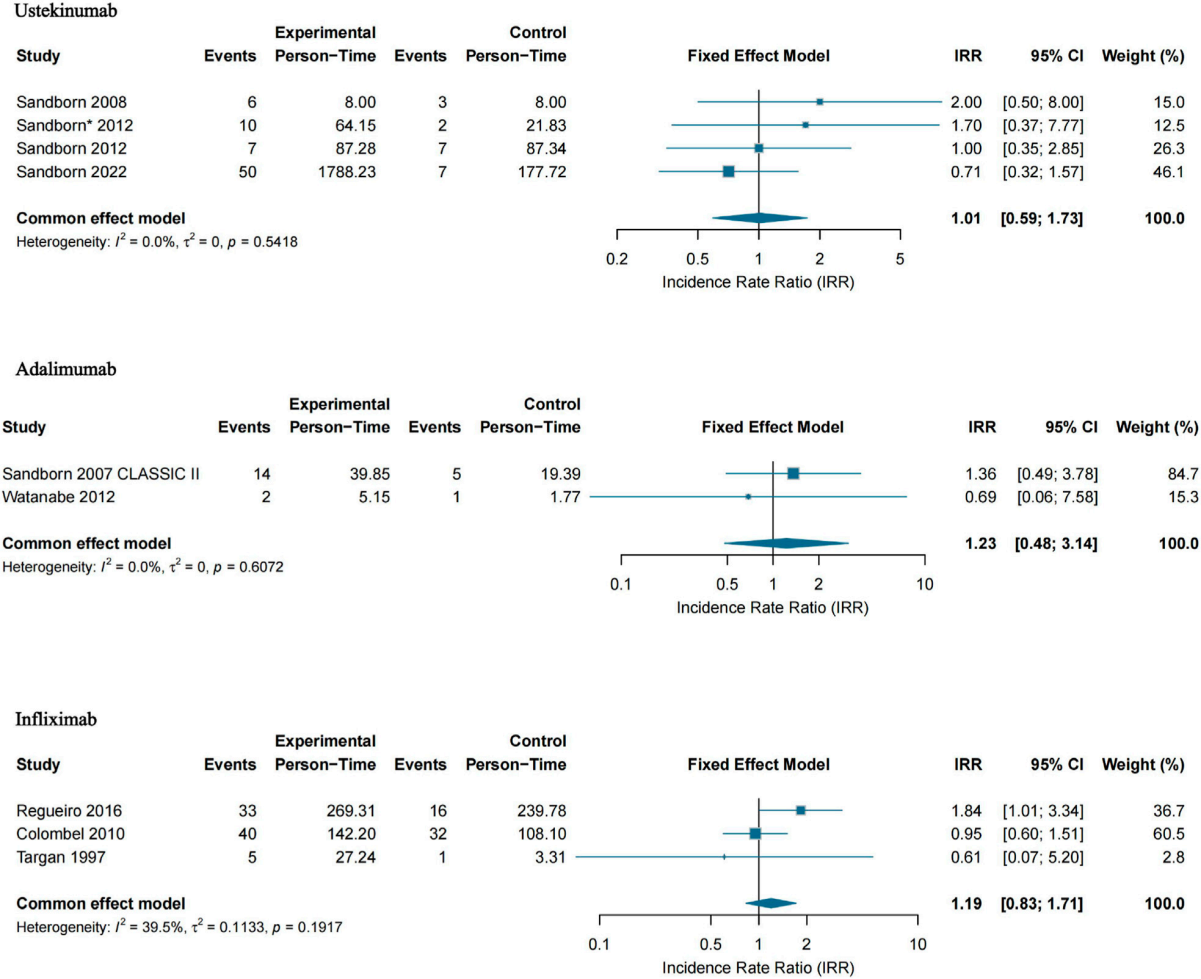
Forest plot illustrating IRR of ustekinumab, adalimumab and infliximab on skin disease vs. placebo. CI confidence interval.

In the pooled analysis, no statistically significant difference was found between UST and placebo in the risk of skin manifestations (IRR 1.01, 95% CI 0.59–1.73, low certainty evidence; Figure 5; Supplementary Table S7). Both ADA and IFX were associated with no statistically significant difference in the risk of skin manifestations compared to placebo (ADA IRR 1.23, 95% CI 0.48–3.14, very low certainty evidence; IFX IRR 1.19, 95% CI 0.83–1.71, low certainty evidence; Figure 5; Supplementary Table S7).

#### 3.3.6 Ocular manifestations

**FIGURE 6:**
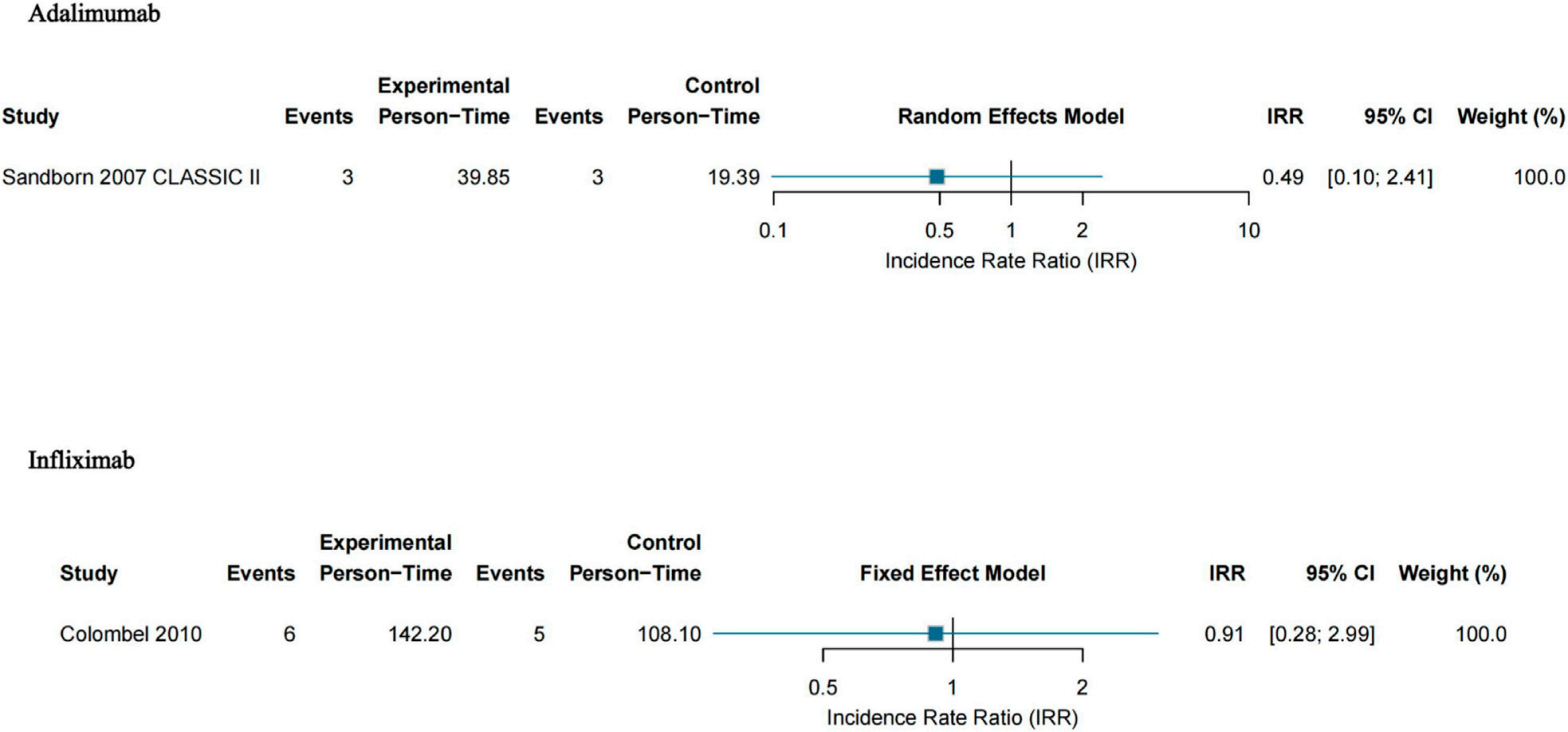
Forest plot illustrating IRR of adalimumab and infliximab on ocular manifestations vs. placebo. CI confidence interval.

The CLASSIC-II maintenance trial (Sandborn et al., 2007a) reported no statistically significant difference in the risk of ocular manifestations between ADA and placebo (IRR 0.49, 95% CI 0.10–2.41, very low certainty evidence; Figure 6; Supplementary Table S8). The SONIC trial (Colombel et al., 2010) reported ocular manifestations in patients treated with IFX. In this trial, no significant difference in the risk of ocular manifestations was found between IFX and placebo (IRR 0.91, 95% CI 0.28–2.99, low certainty evidence; Figure 6; Supplementary Table S8).

#### 3.3.7 Subgroup analysis

A subgroup analysis of ADA at different doses was conducted. No statistically significant difference was found in the risk of fatigue, headache, or arthritis/arthralgia between the standard ADA regimen (every 2 weeks) and placebo (Supplementary Figure S4). However, in the CHARM trial (Colombel et al., 2007), weekly ADA treatment was significantly associated with an increased risk of fatigue (IRR 2.79, 95% CI 1.12–6.94, P = 0.028, moderate quality evidence; Supplementary Figure S4; Supplementary Table S9). For UST and IFX, induction and maintenance subgroup analyses were performed for fatigue, headache, and arthritis/arthralgia. No statistically significant difference was found between the drug and placebo for induction treatment (Supplementary Figure S5). Similarly, no statistically significant difference was observed between IFX and placebo during maintenance treatment with respect to fatigue or headache (Supplementary Figure S6). For maintenance treatment, UST significantly reduced the risk of arthritis/arthralgia (IRR 0.56, 95% CI 0.36–0.86, P = 0.008, low quality evidence; Supplementary Figure S7; Supplementary Table S10), but no significant differences were observed in the risks of fatigue or headache (Supplementary Figure S7).

### 3.4 Safety

In the adverse event assessment, no statistically significant difference was observed between drug treatments and placebo for overall adverse events (Supplementary Figure S8). However, drug treatments were significantly associated with a reduction in the risk of serious adverse events (Supplementary Figure S9).

### 3.5 Sensitivity analysis

Due to low heterogeneity across studies, a leave-one-out sensitivity analysis was performed for the primary outcomes, yielding results consistent with the main analysis.

## 4 Discussion

To our knowledge, this study provides the first comprehensive summary of the impact of UST and TNF antagonists on the risk of EIMs in CD. Overall, we found that UST was associated with a reduced risk of fatigue and arthritis/arthralgia. In contrast, ADA and IFX did not show significant benefits for the prespecified EIMs and, in some cases, demonstrated a tendency to increase risk, particularly with high-dose ADA, which was associated with an increased risk of fatigue. These findings suggest that different biologics may exert differential effects on EIMs, potentially due to mechanistic variations. Among them, UST may play a broader role in modulating systemic inflammatory responses due to its unique target (Murphy et al., 2003). However, as these comparisons were not based on head-to-head trials, they do not provide evidence that UST is superior to ADA or IFX.

Fatigue, one of the most common EIMs of CD, severely affects patients’ quality of life (Lenti et al., 2023). Biologically, fatigue can be considered a major component of the “disease behavior response,” involving the activation of pro-inflammatory cytokines and neuronal biomolecules in the brain (Hart, 1988; D et al., 2014). In our study, UST reduced the risk of fatigue by 47%, and subgroup analysis further supported a positive trend during the maintenance phase. This effect may be explained by UST-mediated inhibition of the IL-12/IL-23 signaling pathways, which reduces the release of pro-inflammatory cytokines such as TNF-α, IL-1β, and IL-6. These cytokines can affect the central nervous system and contribute to fatigue (Irwin, 2008). Additionally, the IL-23 pathway plays a key role in Th17 cell differentiation, and Th17-mediated inflammation has been implicated in the pathophysiology of chronic fatigue syndrome (Broderick et al., 2012). Interestingly, recent studies have shown that anti-IL-23p19 antibodies, such as risankizumab and mirikizumab, can also improve fatigue in CD, further supporting the significance of the IL-23 pathway in the development of fatigue (Chung et al., 2025; Regueiro et al., 2025). By contrast, ADA did not show significant improvements in fatigue at standard doses, and high-dose therapy was associated with an increased risk of fatigue. Some studies suggest that serum concentrations of IFX and ADA, along with anti-drug antibody levels, are unrelated to the severity of fatigue, indicating that the improvement in fatigue with TNF antagonists may mainly stem from their control of intestinal inflammation rather than direct effects on fatigue (Grimstad et al., 2025). Furthermore, attention should be given to high-dose anti-TNF therapy, as it may induce or worsen certain EIMs through paradoxical reactions (Garcovich et al., 2019). It is noteworthy that even with optimal biologic therapy for IBD, many patients in remission still experience persistent fatigue, highlighting the involvement of other pathways in fatigue development (Borren et al., 2020). Two factors consistently linked to fatigue are pain and depression, making early intervention crucial in reducing the long-term burden of fatigue (Staud, 2012).

With respect to joint manifestations, previous studies have reported novel articular outcomes, and UST has been shown to exert a protective effect against arthritis/arthralgia during the maintenance phase, consistent with findings from a cohort study (De Galan et al., 2022). UST may improve joint manifestations by regulating immune pathways shared by the gut and joints through inhibition of IL-12 and IL-23 activity (Kavana et al., 2016). Elevated serum IL-23 levels have been observed in IBD, particularly in CD patients with arthritis and sacroiliitis (Gheita et al., 2014). However, other research has indicated that UST may be less effective in axial spondyloarthritis (Deodhar et al., 2019). For IFX and ADA, no significant difference in the risk of arthritis/arthralgia was observed compared with placebo. However, ADA showed a trend toward increased risk, although this did not reach statistical significance and therefore warrants further attention. This pattern is consistent with clinical practice, in which some patients develop or experience worsening joint symptoms during anti-TNF therapy (Drosos et al., 2021). Potential mechanisms include the dual immunomodulatory role of TNF-α and drug-specific immunogenicity (Gogulescu et al., 2024; Ceglédi et al., 2024). At the same time, it is important to note that TNF antagonists are recommended for axial spondyloarthritis (Ward et al., 2016).

For other prespecified EIM risks, no significant differences were observed between UST and TNF antagonists. Regarding skin manifestations, UST showed potential benefits for CD patients with concomitant pyoderma gangrenosum or erythema nodosum (Nunes et al., 2019; Miranda et al., 2021). But evidence supporting its efficacy for other cutaneous lesions, such as hidradenitis suppurativa, remains inconsistent (Martora et al., 2025). ADA has been reported to successfully treat pyoderma gangrenosum and psoriasiform skin lesions associated with CD, though it may also induce alopecia or lichenoid eruptions (Fousekis et al., 2025; Matucci-Cerinic et al., 2024; Reiss-Huss et al., 2025; Alqefari et al., 2025). With regard to ocular manifestations, TNF antagonists have been shown to be effective in most cases of uveitis, and UST may also have potential benefit for ocular symptoms (Felten and Rosine, 2022). As for anemia, some studies have suggested that adequate post-surgery UST drug levels are associated with a lower risk of anemia (Moskow et al., 2024). In contrast, IFX has been linked to anemia as a predictor of disease progression (Magro et al., 2023).

Several limitations should be acknowledged in our analysis. First, we did not include improvements or worsening of pre-existing EIMs, as comprehensive reviews of this topic already exist (Tímár et al., 2024). Second, the occurrence of EIMs was not the primary endpoint in the included studies; instead, these events were reported as adverse events. Because standardized definitions for EIMs were lacking, most studies did not conduct systematic assessments, which may have introduced measurement bias. Nevertheless, since both investigators and participants were blinded to treatment allocation, such misclassification and underreporting were likely to affect both groups similarly, thereby minimizing the impact on relative comparisons. Third, although heterogeneity across studies was low, we were unable to assess potential confounding effects due to baseline differences, as only summary-level data were available. Fourth, the follow-up duration in the included studies was relatively short, limiting our ability to evaluate long-term risks. Finally, further well-designed RCTs are needed to specifically assess the risk of EIMs associated with biologic therapies in CD. These trials should have sufficiently long follow-up periods to clarify long-term risks. While our study revealed relative associations between biologic therapies and EIMs, the absolute effects may still have been underestimated due to reporting limitations.

## 5 Conclusion

Existing data show that UST significantly reduces the risk of fatigue and arthritis/arthralgia in CD patients, while high-dose ADA is associated with an increased risk of fatigue. UST may be considered for CD patients at high risk of fatigue and arthritis/arthralgia. It may remain a clinically relevant treatment option, particularly for patients requiring alternatives to anti-TNF therapy, although dedicated studies on EIM-specific outcomes are still needed. Future rigorously designed clinical trial are needed to assess the efficacy of biologics in treating multiple EIMs, thereby improving overall treatment outcomes in CD.

## Data Availability

The original contributions presented in the study are included in the article/Supplementary Material, further inquiries can be directed to the corresponding authors.
