# Supplementary material for: The impact of ustekinumab and tumor necrosis factor antagonists on extraintestinal manifestations of Crohn’s disease: a systematic review and meta-analysis
Source: Front Pharmacol. 2026 Jul 30;17:1849397. doi: 10.3389/fphar.2026.1849397 (PMC13467844; doi:10.3389/fphar.2026.1849397)
Supplement: Supplementary file 1 [file DataSheet1.pdf]

Online Supplementary Material

Title: The Impact of Ustekinumab and Tumor Necrosis Factor Inhibitors on Extraintestinal Manifestations of Crohn's Disease: A Systematic Review and Meta-Analysis

Supplementary Table 1 Search strategy.

Supplementary Table 2 Outcome data on extraintestinal manifestations in the included trials.

Supplementary Table 3 Ustekinumab, adalimumab and infliximab compared to placebo for fatigue risk in patients with moderately to severely active Crohn's disease.

Supplementary Table 4 Ustekinumab, adalimumab and infliximab compared to placebo for headache risk in patients with moderately to severely active Crohn's disease.

Supplementary Table 5 Ustekinumab, adalimumab and infliximab compared to placebo for arthritis/arthralgia risk in patients with moderately to severely active Crohn's disease.

Supplementary Table 6 Ustekinumab and infliximab compared to placebo for anaemia risk in patients with moderately to severely active Crohn's disease.

Supplementary Table 7 Ustekinumab, adalimumab and infliximab compared to placebo for skin disease risk in patients with moderately to severely active Crohn's disease.

Supplementary Table 8 Adalimumab and infliximab compared to placebo for ocular manifestations risk in patients with moderately to severely active Crohn's disease.

Supplementary Table 9 Adalimumab compared to placebo for fatigue risk in patients with moderately to severely active Crohn's disease in subgroup analyses.

Supplementary Table 10 Ustekinumab compared to placebo for arthritis/arthralgia risk in patients with moderately to severely active Crohn's disease in subgroup analyses.

Supplementary Figure 1 PRISMA [Preferred Reporting Items for Systematic Reviews and Meta-Analyses] flowchart.

Supplementary Figure 2 Results of RoB 2 assessment of the included studies.

Supplementary Figure 3 Results of RoB 2 assessment of the included studies.

Supplementary Figure 4 Forest plot illustrating IRR of adalimumab on fatigue, headache and arthritis/arthralgia vs. placebo in subgroup analyses.

Supplementary Figure 5 A Forest plot illustrating IRR of ustekinumab on fatigue, headache and arthritis/arthralgia vs. placebo in subgroup analyses. **B** Forest plot illustrating IRR of infliximab on fatigue and headache vs. placebo in subgroup analyses.

Supplementary Figure 6 Forest plot illustrating IRR of infliximab on fatigue and

headache vs. placebo in subgroup analyses.

Supplementary Figure 7 Forest plot illustrating IRR of ustekinumab on fatigue, headache and arthritis/arthralgia vs. placebo in subgroup analyses.

Supplementary Figure 8 Forest plot illustrating RR of ustekinumab, adalimumab and infliximab on adverse event vs. placebo.

Supplementary Figure 9 Forest plot illustrating IRR of ustekinumab, adalimumab and infliximab on serious adverse event vs. placebo

Supplementary Table 1 Search strategy.

Ustekinumab OR CNTO 1275 OR CNTO-1275 OR Stelara
OR
Adalimumab OR Humira OR Amjevita OR Cyltezo OR D2E7 Antibody OR Antibody，D2E7 OR Adalimumab-atto OR Adalimumab-adbm
OR
Infliximab OR Monoclonal Antibody cA2 OR Antibody cA2, Monoclonal OR cA2, Monoclonal Antibody OR MAb cA2 OR Remicade OR Inflectra OR Renflexis OR Infliximab-dyyb OR Infliximab dyyb OR Infliximab-abda OR Infliximab abda
AND
Crohn's Disease OR Crohns Disease OR Crohn's Enteritis OR Inflammatory Bowel Disease 1 OR Regional Enteritis OR Ileocolitis OR Ileitis, Terminal OR Terminal Ileitis OR Ileitis, Regional OR Regional Ileitides OR Regional Ileitis OR Enteritis, Granulomatous OR Granulomatous Enteritis OR Enteritis, Regional OR Colitis, Granulomatous OR Granulomatous Colitis

Note – extraintestinal manifestations terms are intentionally omitted as they are unlikely to be included in study abstracts.

Supplementary Table 2 Outcome data on extraintestinal manifestations in the included trials.

Outcome data of Included Trials

		Extraintestinal manifestations/envents							
**Source**	**Intervention**	**Fatigue[a]**	**Headache[a]**	**Arthritis/arthralgia[a]**	**Anaemia[a]**	**Skin disease[a]**	**Ocular manifestations[a]**	**Adverse events[b]**	**Serious adverse event[b]**
**Adalimumab**									
Colombel[36], 2007 CHARM	Placebo	6	15	23	N/A	N/A	N/A	211	40
	Adalimumab	31	55	61	N/A	N/A	N/A	451	45
Rutgeerts[37], 2012 EXTEND	Placebo	2	6	4	N/A	N/A	N/A	55	5
	Adalimumab	7	9	7	N/A	N/A	N/A	61	4
Sandborn[38], 2007 CLASSIC II	Placebo	4	3	1	N/A	5	3	18	2
	Adalimumab	3	0	3	N/A	14	3	29	1
Chen[39], 2020	Placebo	N/A	N/A	N/A	N/A	N/A	N/A	18	1
	Adalimumab	N/A	N/A	N/A	N/A	N/A	N/A	25	2
Watanabe[40], 2014	Placebo	N/A	2	N/A	N/A	N/A	N/A	13	6
	Adalimumab	N/A	1	N/A	N/A	N/A	N/A	17	2
Sandborn[41], 2007	Placebo	9	12	3	N/A	N/A	N/A	121	8
	Adalimumab	7	8	9	N/A	N/A	N/A	91	2
Watanabe[42], 2012	Placebo	N/A	1	N/A	N/A	1	N/A	8	2
	Adalimumab	N/A	3	N/A	N/A	2	N/A	27	4
Hanauer[43], 2006 CLASSIC-I	Placebo	N/A	4	N/A	N/A	N/A	N/A	55	3
	Adalimumab	N/A	14	N/A	N/A	N/A	N/A	158	4

**Ustekinumab**									
Feagan[16], 2016 UNITI-1	Placebo	13	22	19	N/A	N/A	N/A	90	22
	Ustekinumab	16	40	43	N/A	N/A	N/A	173	45
Feagan[16], 2016 UNITI-2	Placebo	N/A	14	N/A	N/A	N/A	N/A	44	19
	Ustekinumab	N/A	30	N/A	N/A	N/A	N/A	84	24
Sandborn[44], 2008 C0379T07	Placebo	6	8	3	N/A	3	N/A	41	3
	Ustekinumab	1	6	3	N/A	6	N/A	37	5
Sandborn[45], 2012 CERTIFI	Placebo	9	19	19	N/A	9	N/A	N/A	N/A
	Ustekinumab	11	42	33	N/A	17	N/A	N/A	N/A
Sandborn[46], 2022 IM-UNITI	Placebo	8	12	12	2	7	N/A	83	36
	Ustekinumab	42	59	72	32	50	N/A	455	277
Ferrante[47], 2024 VIVID-1	Placebo	N/A	6	10	10	N/A	N/A	66	48
	Ustekinumab	N/A	15	8	15	N/A	N/A	131	48
**Infliximab**									
Hanauer[48], 2024 LIBERTY-CD	Placebo	N/A	5	N/A	5	N/A	N/A	30	8
	infliximab	N/A	18	N/A	13	N/A	N/A	75	16
Regueiro[49], 2016	Placebo	18	34	32	10	16	N/A	122	36
	Infliximab	16	37	33	8	33	N/A	147	39
Present[50], 1999	Placebo	2	7	N/A	N/A	N/A	N/A	20	33
	Infliximab	6	11	N/A	N/A	N/A	N/A	41	19
Sands[51], 2004	Placebo	N/A	N/A	N/A	N/A	N/A	N/A	132	N/A
	Infliximab	N/A	N/A	N/A	N/A	N/A	N/A	123	N/A
Rutgeerts[52],	Placebo	N/A	4	N/A	N/A	N/A	N/A	35	N/A

1999	Infliximab	N/A	6	N/A	N/A	N/A	N/A	35	N/A
Regueiro[53], 2009	Placebo	N/A		2	N/A	N/A	N/A	11	N/A
	Infliximab	N/A	N/A	0	N/A	N/A	N/A	7	N/A
Hanauer[54], 2002 ACCENT I	Placebo	N/A	N/A	N/A	N/A	N/A	N/A	211	55
	Infliximab	N/A	N/A	N/A	N/A	N/A	N/A	451	97
Colombel[55], 2010 SONIC	Placebo	25	20	18	6	32	5	144	43
	Infliximab	26	23	21	3	40	6	161	27
Targan[56], 1997	Placebo	1	5	N/A	N/A	1	N/A	15	N/A
	Infliximab	9	22	N/A	N/A	5	N/A	99	N/A

[a] Number of times events occurred during the trial.

[b] Number of patients in whom an event, occurred ≧1 time during the trial.

Supplementary Table 3 Ustekinumab, adalimumab and infliximab compared to placebo for fatigue risk in patients with moderately to severely active Crohn's disease.

Patient or population: patients with moderately to severely active CD
Setting:
Intervention: ustekinumab, adalimumab and infliximab
Comparison: placebo

Outcomes	Anticipated absolute effects* (95% CI) / PY		Relative effect (95% CI)	PYs (studies)	Certainty of the evidence (GRADE)
	Risk with placebo	Risk with drugs			
fatigue risk (ustekinumab)	108 per 1,000	57 per 1,000 (36 - 91)	IRR 0.53 (0.33 - 0.84)	2360.70 (5 RCTs)	⊕⊕⊕◯ Moderate[a]
fatigue risk (adalimumab)	79 per 1,000	92 per 1,000 (42 - 205)	IRR 1.17 (0.53 - 2.59)	837.31 (4 RCTs)	⊕◯◯◯ Very low[b]
fatigue risk (infliximab)	127 per 1,000	105 per 1,000 (71 - 159)	IRR 0.83 (0.56 - 1.25)	822.48 (4 RCTs)	⊕⊕◯◯ Low[c]

***The risk in the intervention group** (and its 95% confidence interval) is based on the assumed risk in the comparison group and the **relative effect** of the intervention (and its 95% CI).
CI: confidence interval; **IRR:** incidence rate ratios; **PY:** patients year

GRADE Working Group grades of evidence
High certainty: we are very confident that the true effect lies close to that of the estimate of the effect.
Moderate certainty: we are moderately confident in the effect estimate: the true effect is likely to be close to the estimate of the effect, but there is a possibility that it is substantially different.
Low certainty: our confidence in the effect estimate is limited: the true effect may be substantially different from the estimate of the effect.
Very low certainty: we have very little confidence in the effect estimate: the true effect is likely to be substantially different from the estimate of effect.

Explanations
a. Downgraded one level due to sparse data (106 events)
b. Downgraded three levels due to extremely serious imprecision (69 events)
c. Downgraded two levels due to imprecision and sparse data (103 event)

Supplementary Table 4 Ustekinumab, adalimumab and infliximab compared to placebo for headache risk in patients with moderately to severely active Crohn's disease.

Patient or population: patients with moderately to severely active CD
Setting:
Intervention: ustekinumab, adalimumab and infliximab
Comparison: placebo

Outcomes	Anticipated absolute effects* (95% CI) / PY		Relative effect (95% CI)	PYs (studies)	Certainty of the evidence (GRADE)
	Risk with placebo	Risk with drugs			
headache risk (ustekinumab)	167 per 1,000	147 per 1,000 (112 - 192)	IRR 0.88 (0.67 - 1.15)	2871.80 (7 RCTs)	⊕⊕⊕◯ Moderate[a]
headache risk (adalimumab)	149 per 1,000	165 per 1,000 (112 - 244)	IRR 1.11 (0.75 - 1.64)	896.22 (7 RCTs)	⊕⊕◯◯ Low[b]
headache risk (infliximab)	159 per 1,000	148 per 1,000 (110 - 213)	IRR 0.93 (0.69 - 1.34)	1155.2 (6 RCTs)	⊕⊕⊕◯ Moderate[c]

***The risk in the intervention group** (and its 95% confidence interval) is based on the assumed risk in the comparison group and the relative effect of the intervention (and its 95% CI).
CI: confidence interval; **IRR:** incidence rate ratios; **PY:** patients year

GRADE Working Group grades of evidence
High certainty: we are very confident that the true effect lies close to that of the estimate of the effect.
Moderate certainty: we are moderately confident in the effect estimate: the true effect is likely to be close to the estimate of the effect, but there is a possibility that it is substantially different.
Low certainty: our confidence in the effect estimate is limited: the true effect may be substantially different from the estimate of the effect.
Very low certainty: we have very little confidence in the effect estimate: the true effect is likely to be substantially different from the estimate of effect.

Explanations
a. Downgraded one level due to imprecision (273 events)
b. Downgraded two levels due to serious imprecision (133 events)
c. Downgraded one level due to imprecision (192 events)

Supplementary Table 5 Ustekinumab, adalimumab and infliximab compared to placebo for arthritis/arthralgia risk in patients with moderately to severely active Crohn's disease.

Patient or population: patients with moderately to severely active CD
Setting:
Intervention: ustekinumab, adalimumab and infliximab
Comparison: placebo

Outcomes	Anticipated absolute effects* (95% CI) / PY		Relative effect (95% CI)	PYs (studies)	Certainty of the evidence (GRADE)
	Risk with placebo	Risk with drugs			
arthritis/arthralgia risk (ustekinumab)	139 per 1,000	110 per 1,000 (71 - 170)	IRR 0.79 (0.51 - 1.22)	2773.5 (6 RCTs)	⊕⊕⊕◯ Moderate[a]
arthritis/arthralgia risk (adalimumab)	117 per 1,000	150 per 1,000 (98 - 227)	IRR 1.28 (0.84 - 1.94)	837.31 (4 RCTs)	⊕◯◯◯ Very low[b]
arthritis/arthralgia risk (infliximab)	144 per 1,000	128 per 1,000 (88 - 189)	IRR 0.89 (0.61 - 1.31)	783.39 (3 RCTs)	⊕⊕◯◯ Low[c]

***The risk in the intervention group** (and its 95% confidence interval) is based on the assumed risk in the comparison group and the relative effect of the intervention (and its 95% CI).
CI: confidence interval; **IRR:** incidence rate ratios; **PY:** patients year

GRADE Working Group grades of evidence
High certainty: we are very confident that the true effect lies close to that of the estimate of the effect.
Moderate certainty: we are moderately confident in the effect estimate: the true effect is likely to be close to the estimate of the effect, but there is a possibility that it is substantially different.
Low certainty: our confidence in the effect estimate is limited: the true effect may be substantially different from the estimate of the effect.
Very low certainty: we have very little confidence in the effect estimate: the true effect is likely to be substantially different from the estimate of effect.

Explanations
a. Downgraded one level due to imprecision (222 events)
b. Downgraded three levels due to extremely serious imprecision (111 events)
c. Downgraded two levels due to sparse data and imprecision (106 events)

Supplementary Table 6 Ustekinumab and infliximab compared to placebo for anaemia risk in patients with moderately to severely active Crohn's disease.

Patient or population: patients with moderately to severely active CD
Setting:
Intervention: ustekinumab and infliximab
Comparison: placebo

Outcomes	Anticipated absolute effects* (95% CI) / PY		Relative effect (95% CI)	PYs (studies)	Certainty of the evidence (GRADE)
	Risk with placebo	Risk with drugs			
anaemia risk (ustekinumab)	40 per 1,000	61 per 1,000 (30 - 122)	IRR 1.52 (0.76 - 3.06)	2204.95 (2 RCTs)	⊕⊕◯◯ Low[a]
anaemia risk (infliximab)	48 per 1,000	36 per 1,000 (19 - 66)	IRR 0.75 (0.40 - 1.38)	1048.79 (3 RCTs)	⊕⊕◯◯ Low[b]

***The risk in the intervention group** (and its 95% confidence interval) is based on the assumed risk in the comparison group and the relative effect of the intervention (and its 95% CI).
CI: confidence interval; **IRR:** incidence rate ratios; **PY:** patients year

GRADE Working Group grades of evidence
High certainty: we are very confident that the true effect lies close to that of the estimate of the effect.
Moderate certainty: we are moderately confident in the effect estimate: the true effect is likely to be close to the estimate of the effect, but there is a possibility that it is substantially different.
Low certainty: our confidence in the effect estimate is limited: the true effect may be substantially different from the estimate of the effect.
Very low certainty: we have very little confidence in the effect estimate: the true effect is likely to be substantially different from the estimate of effect.

Explanations
a. Downgraded two levels due to serious imprecision (59 events)
b. Downgraded two levels due to serious imprecision (45 events)

Supplementary Table 7 Ustekinumab, adalimumab and infliximab compared to placebo for skin disease risk in patients with moderately to severely active Crohn's disease.

Patient or population: patients with moderately to severely active CD
Setting:
Intervention: ustekinumab, adalimumab and infliximab
Comparison: placebo

Outcomes	Anticipated absolute effects* (95% CI) / PY		Relative effect (95% CI)	PYs (studies)	Certainty of the evidence (GRADE)
	Risk with placebo	Risk with drugs			
skin disease risk (ustekinumab)	64 per 1,000	65 per 1,000 (38 - 111)	IRR 1.01 (0.59 - 1.73)	2242.55 (4 RCTs)	⊕⊕◯◯ Low[a]
skin disease risk (adalimumab)	284 per 1,000	349 per 1,000 (136 - 892)	IRR 1.23 (0.48 - 3.14)	66.16 (2 RCTs)	⊕◯◯◯ Very low[b]
skin disease risk (infliximab)	140 per 1,000	167 per 1,000 (116 - 239)	IRR 1.19 (0.83 - 1.71)	789.94 (3 RCTs)	⊕⊕◯◯ Low[c]

***The risk in the intervention group** (and its 95% confidence interval) is based on the assumed risk in the comparison group and the relative effect of the intervention (and its 95% CI).
CI: confidence interval; **IRR:** incidence rate ratios; **PY:** patients year

GRADE Working Group grades of evidence
High certainty: we are very confident that the true effect lies close to that of the estimate of the effect.
Moderate certainty: we are moderately confident in the effect estimate: the true effect is likely to be close to the estimate of the effect, but there is a possibility that it is substantially different.
Low certainty: our confidence in the effect estimate is limited: the true effect may be substantially different from the estimate of the effect.
Very low certainty: we have very little confidence in the effect estimate: the true effect is likely to be substantially different from the estimate of effect.

Explanations
a. Downgraded two levels due to serious imprecision (92 events)
b. Downgraded three levels due to extremely serious imprecision (22 events)
c. Downgraded one level due to serious imprecision (127 events)

Supplementary Table 8 Adalimumab and infliximab compared to placebo for ocular manifestations risk in patients with moderately to severely active Crohn's disease.

Patient or population: patients with moderately to severely active CD
Setting:
Intervention: adalimumab and infliximab
Comparison: placebo

Outcomes	Anticipated absolute effects* (95% CI) / PY		Relative effect (95% CI)	PYs (studies)	Certainty of the evidence (GRADE)
	Risk with placebo	Risk with drugs			
ocular manifestations risk (adalimumab)	155 per 1,000	76 per 1,000 (16 - 374)	IRR 0.49 (0.10 - 2.41)	59.24 (1 RCTs)	⊕◯◯◯ Very low[a]
ocular manifestations risk (infliximab)	46 per 1,000	42 per 1,000 (13 - 138)	IRR 0.91 (0.28 - 2.99)	250.30 (1 RCTs)	⊕◯◯◯ Very low[b]

***The risk in the intervention group** (and its 95% confidence interval) is based on the assumed risk in the comparison group and the relative effect of the intervention (and its 95% CI).
CI: confidence interval; **IRR:** incidence rate ratios; **PY:** patients year

GRADE Working Group grades of evidence
High certainty: we are very confident that the true effect lies close to that of the estimate of the effect.
Moderate certainty: we are moderately confident in the effect estimate: the true effect is likely to be close to the estimate of the effect, but there is a possibility that it is substantially different.
Low certainty: our confidence in the effect estimate is limited: the true effect may be substantially different from the estimate of the effect.
Very low certainty: we have very little confidence in the effect estimate: the true effect is likely to be substantially different from the estimate of effect.

Explanations
a. Downgraded three levels due to extremely serious imprecision (6 events)
b. Downgraded two levels due to extremely serious sparse data (11 events)

Supplementary Table 9 Adalimumab compared to placebo for fatigue risk in patients with moderately to severely active Crohn's disease in subgroup analyses.

Patient or population: patients with moderately to severely active CD
Setting:
Intervention: adalimumab
Comparison: placebo

Outcomes	Anticipated absolute effects* (95% CI) / PY		Relative effect (95% CI)	PYs (studies)	Certainty of the evidence (GRADE)
	Risk with placebo	Risk with drugs			
fatigue risk (adalimumab)	28 per 1,000	78 per 1,000 (31 - 194)	IRR 2.79 (1.12 - 6.94)	464.10 (1 RCTs)	⊕⊕⊕◯ Moderate[a]

***The risk in the intervention group** (and its 95% confidence interval) is based on the assumed risk in the comparison group and the relative effect of the intervention (and its 95% CI).
CI: confidence interval; **IRR:** incidence rate ratios; **PY:** patients year

GRADE Working Group grades of evidence
High certainty: we are very confident that the true effect lies close to that of the estimate of the effect.
Moderate certainty: we are moderately confident in the effect estimate: the true effect is likely to be close to the estimate of the effect, but there is a possibility that it is substantially different.
Low certainty: our confidence in the effect estimate is limited: the true effect may be substantially different from the estimate of the effect.
Very low certainty: we have very little confidence in the effect estimate: the true effect is likely to be substantially different from the estimate of effect.

Explanations
a. Downgraded one level due to sparse data (26 events)

Supplementary Table 10 Ustekinumab compared to placebo for arthritis/arthralgia risk in patients with moderately to severely active Crohn's disease in subgroup analyses.

Patient or population: patients with moderately to severely active CD
Setting:
Intervention: ustekinumab
Comparison: placebo

Outcomes	Anticipated absolute effects* (95% CI) / PY		Relative effect (95% CI)	PYs (studies)	Certainty of the evidence (GRADE)
	Risk with placebo	Risk with drugs			
arthritis/arthralgia risk (adalimumab)	96 per 1,000	54 per 1,000 (35 - 83)	IRR 0.56 (0.36 - 0.86)	2553.37 (3 RCTs)	⊕⊕◯◯ Low[a]

***The risk in the intervention group** (and its 95% confidence interval) is based on the assumed risk in the comparison group and the relative effect of the intervention (and its 95% CI).
CI: confidence interval; **IRR:** incidence rate ratios; **PY:** patients year

GRADE Working Group grades of evidence
High certainty: we are very confident that the true effect lies close to that of the estimate of the effect.
Moderate certainty: we are moderately confident in the effect estimate: the true effect is likely to be close to the estimate of the effect, but there is a possibility that it is substantially different.
Low certainty: our confidence in the effect estimate is limited: the true effect may be substantially different from the estimate of the effect.
Very low certainty: we have very little confidence in the effect estimate: the true effect is likely to be substantially different from the estimate of effect.

Explanations
a. Downgraded two levels due to serious imprecision (128 events)

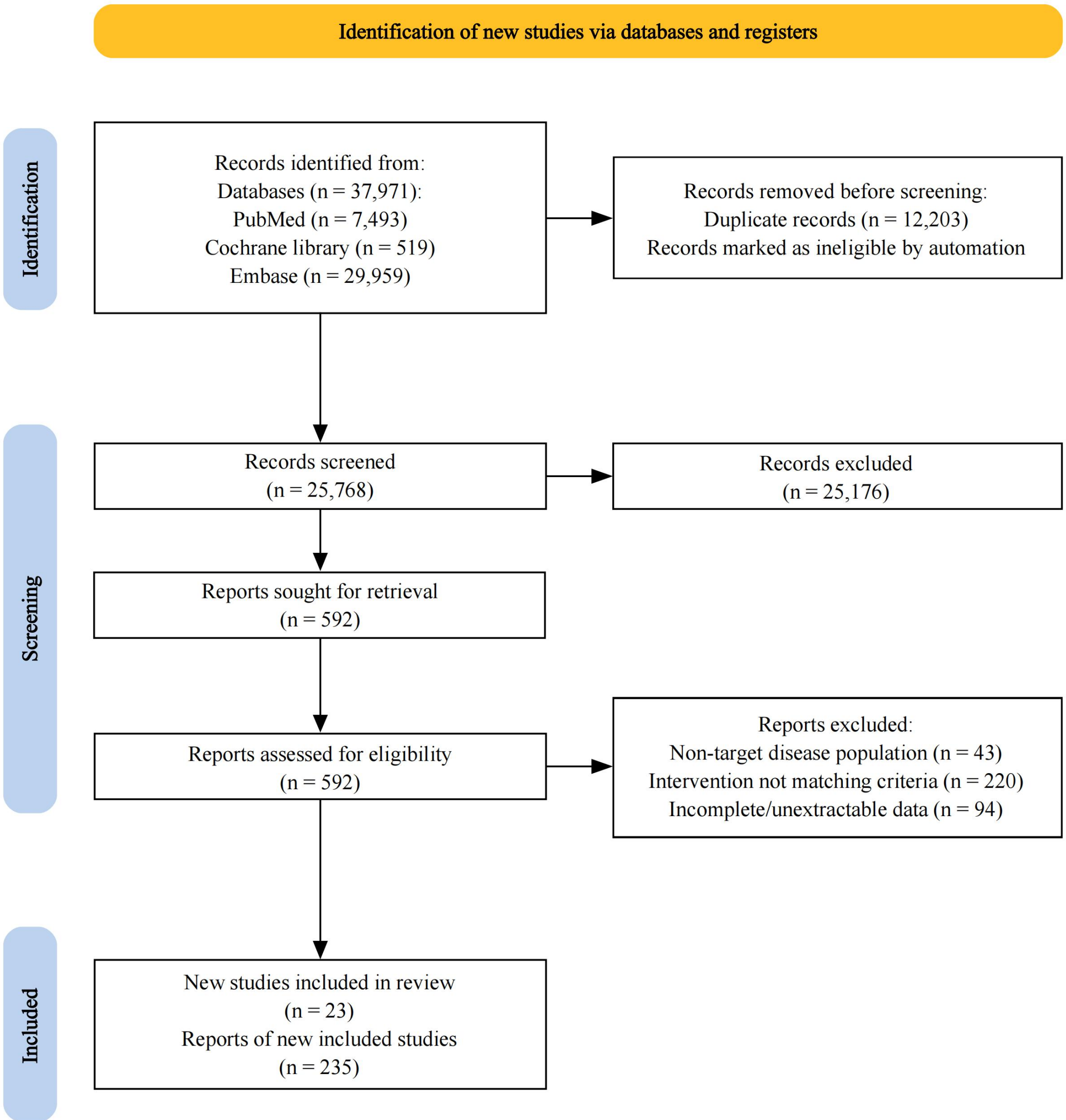

Supplementary Figure 1 PRISMA [Preferred Reporting Items for Systematic Reviews and Meta-Analyses] flowchart.

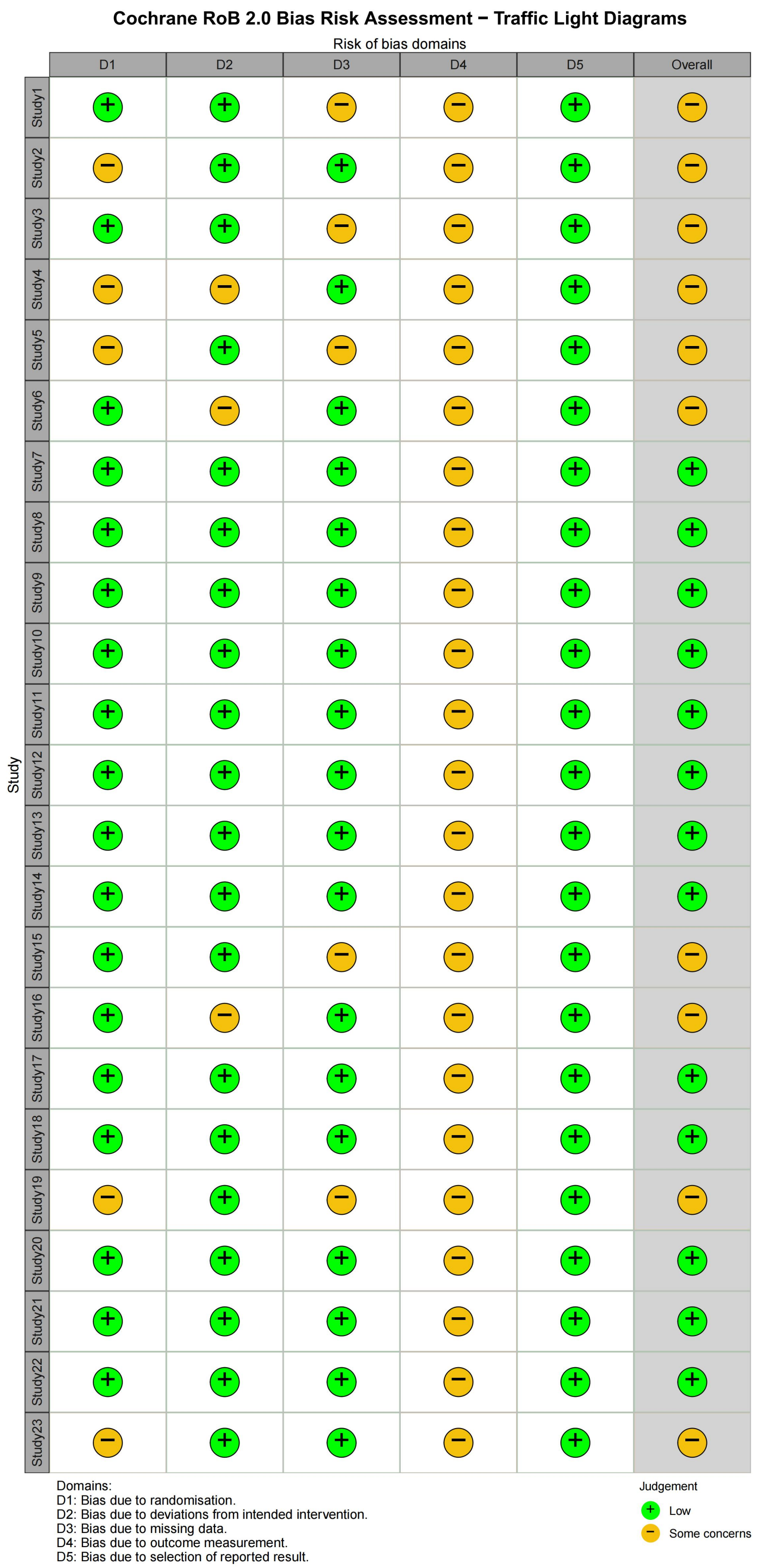

Supplementary Figure 2 Results of RoB 2 assessment of the included studies.

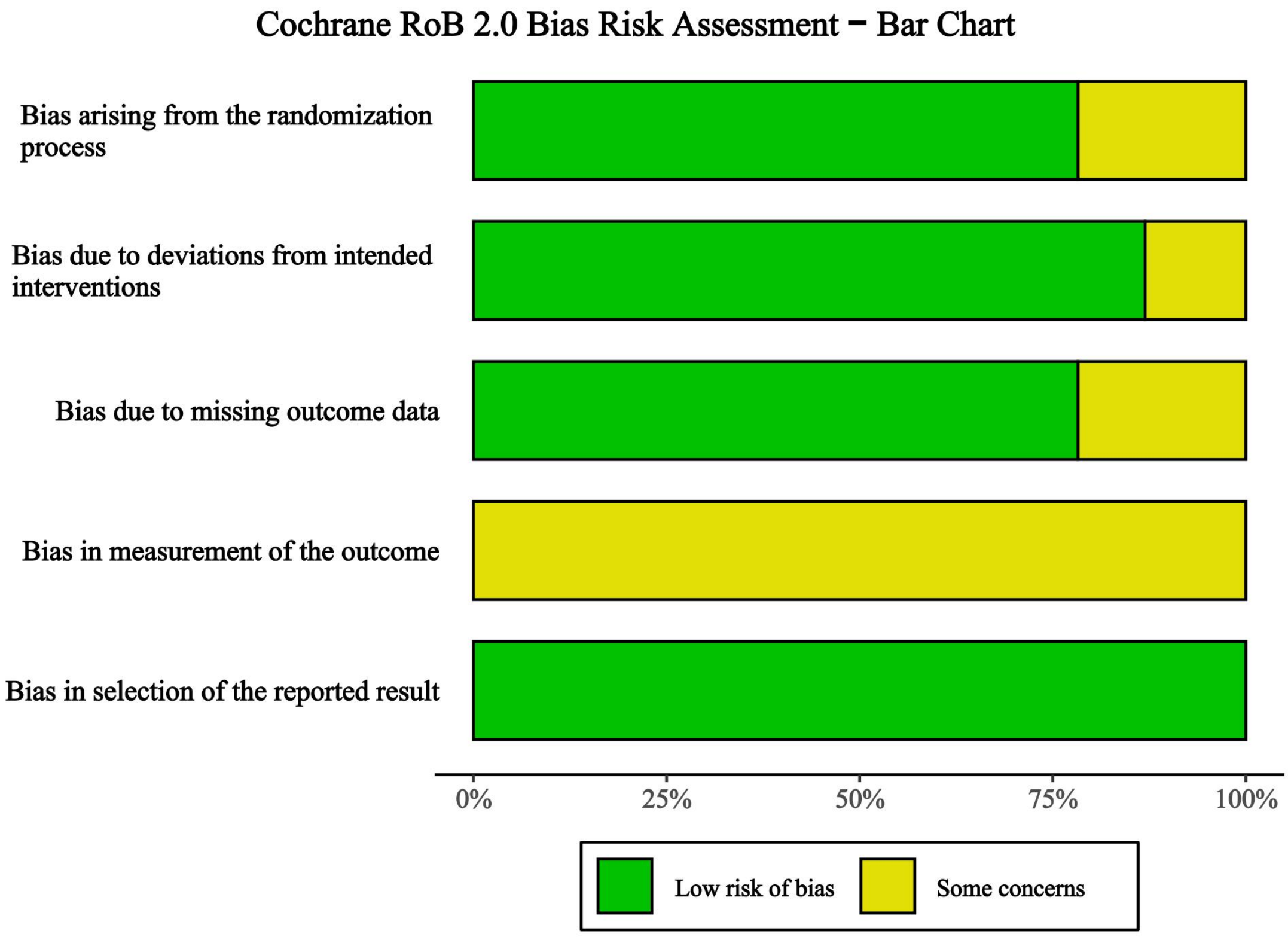

Supplementary Figure 3 Results of RoB 2 assessment of the included studies.

Fatigue

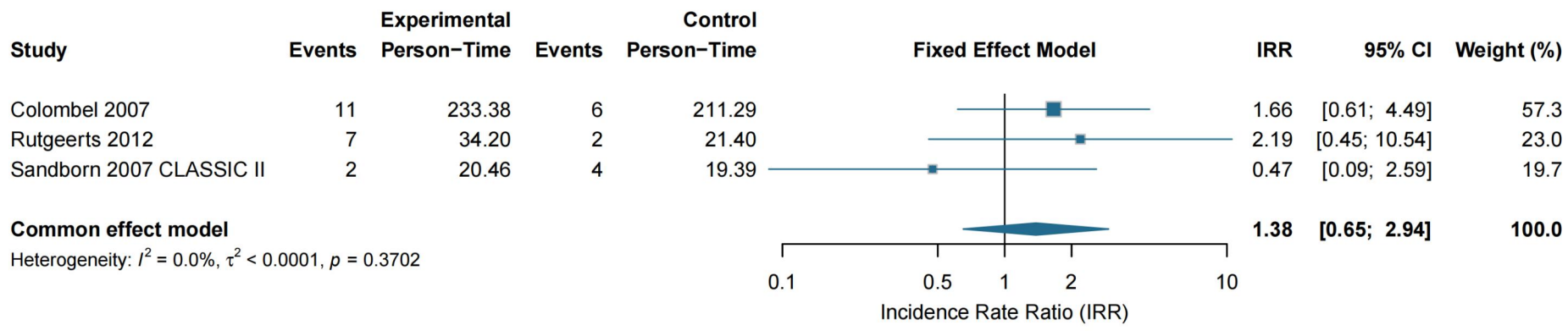

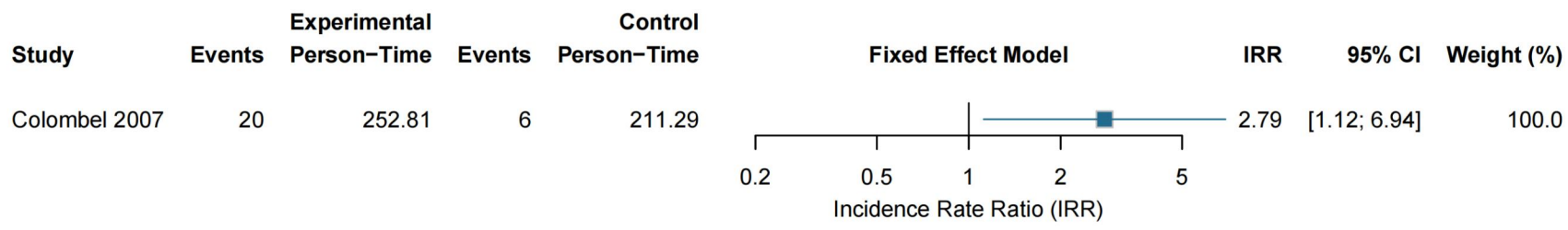

Headache

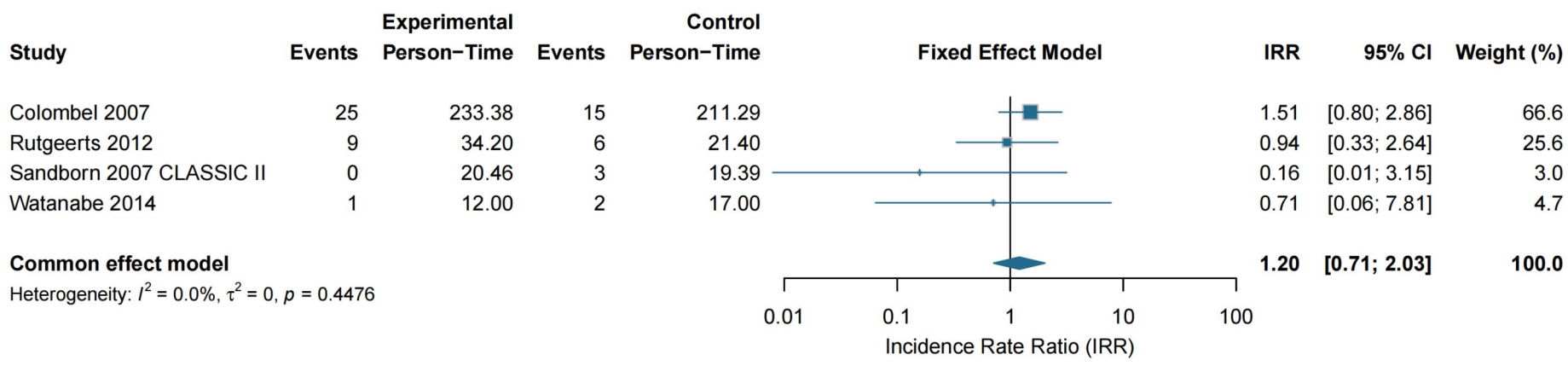

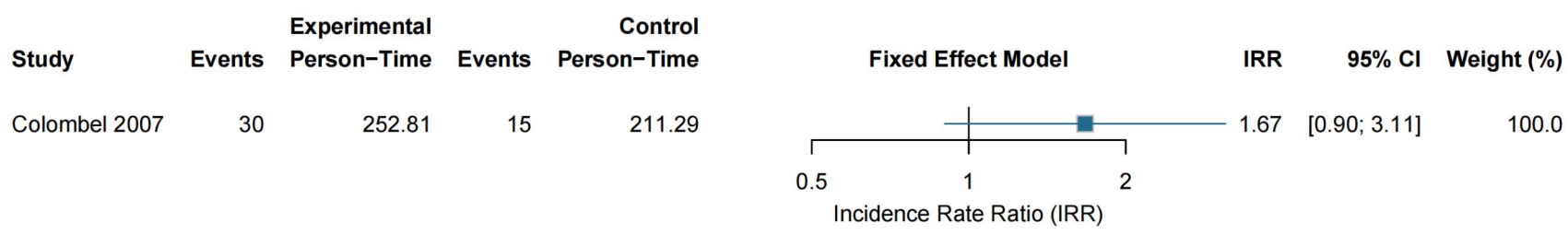

Arthritis/arthralgia

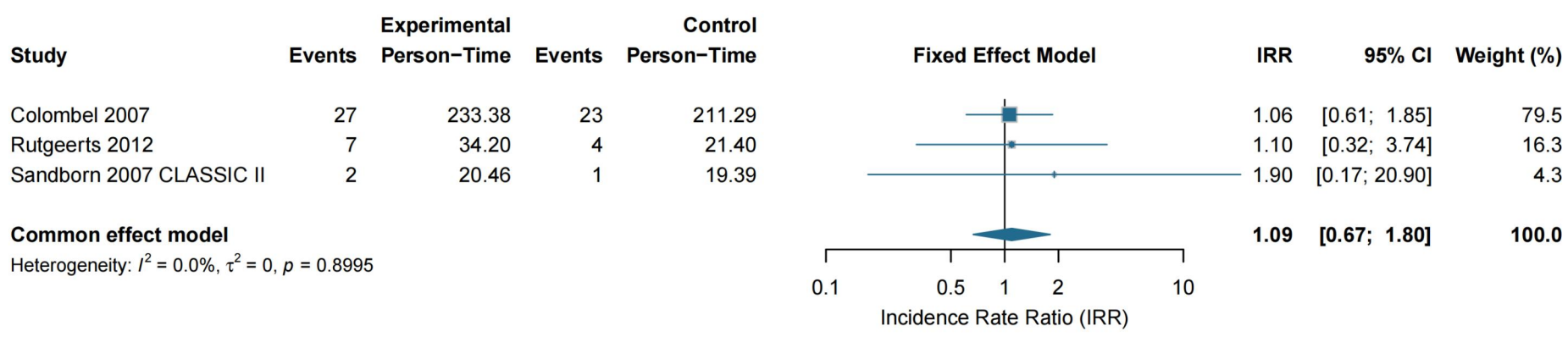

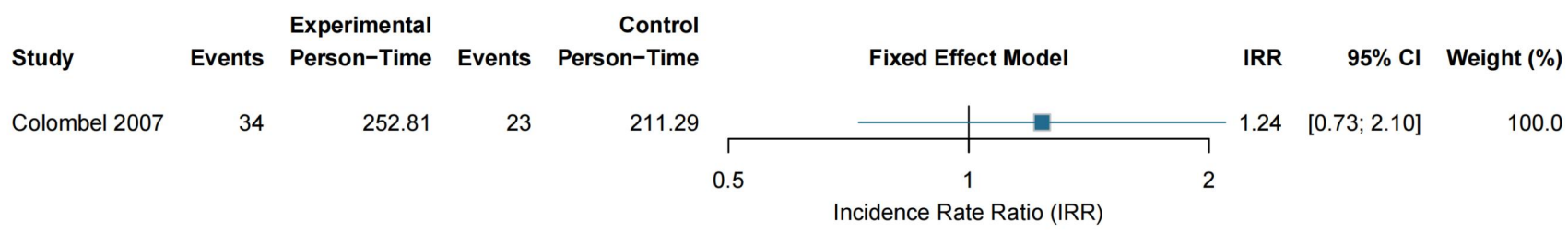

Supplementary Figure 4 Forest plot illustrating IRR of adalimumab on fatigue, headache and arthritis/arthralgia vs. placebo in subgroup analyses. CI confidence interval.

A

Fatigue

Study	Experimental Events	Experimental Person−Time	Control Events	Control Person−Time	IRR	95% CI	Weight (%)
Feagan 2016 UNITI−1	16	79.08	13	39.07	0.61	[0.29; 1.26]	42.1
Sandborn 2008	1	8.00	6	8.00	0.17	[0.02; 1.38]	31.9
Sandborn* 2012	8	87.28	0	87.34	16.01	[0.92; 278.77]	26.0
**Random effects model**					**0.94**	**[0.10; 9.18]**	**100.0**

Heterogeneity: I^2 = 69.0%, τ^2 = 3.0645, p = 0.0398

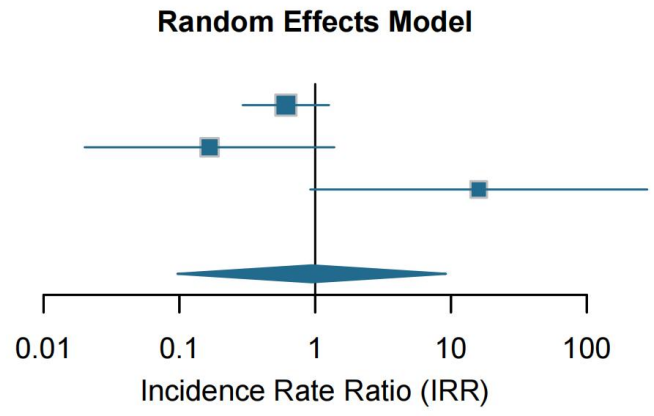

Headache

Study	Experimental Events	Experimental Person−Time	Control Events	Control Person−Time	IRR	95% CI	Weight (%)
Feagan 2016 UNITI−1	40	79.08	22	39.07	0.90	[0.53; 1.51]	42.4
Feagan 2016 UNITI−2	30	65.69	14	32.61	1.06	[0.56; 2.01]	28.5
Sandborn 2008	6	8.00	8	8.00	0.75	[0.26; 2.16]	10.2
Sandborn* 2012	30	64.15	8	21.83	1.28	[0.59; 2.78]	18.9
**Common effect model**					**0.99**	**[0.70; 1.39]**	**100.0**

Heterogeneity: I^2 = 0.0%, τ^2 = 0, p = 0.8363

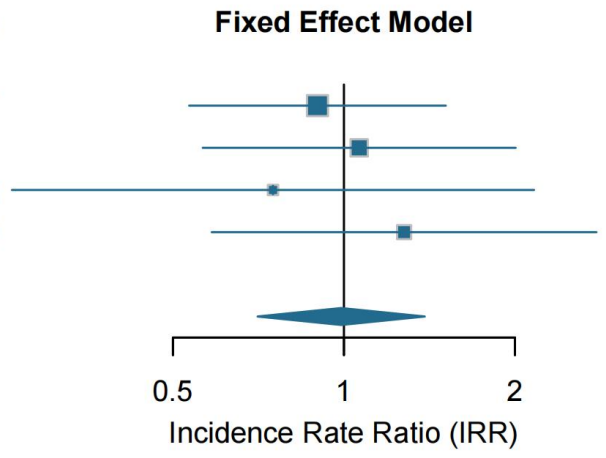

Arthritis/arthralgia

Study	Experimental Events	Experimental Person−Time	Control Events	Control Person−Time	IRR	95% CI	Weight (%)
Feagan 2016 UNITI−1	43	79.08	19	39.07	1.12	[0.65; 1.92]	73.0
Sandborn 2008	3	8.00	3	8.00	1.00	[0.20; 4.95]	8.3
Sandborn* 2012	22	64.15	4	21.83	1.87	[0.64; 5.43]	18.7
**Random effects model**					**1.22**	**[0.77; 1.93]**	**100.0**

Heterogeneity: I^2 = 0.0%, τ^2 = 0, p = 0.6773

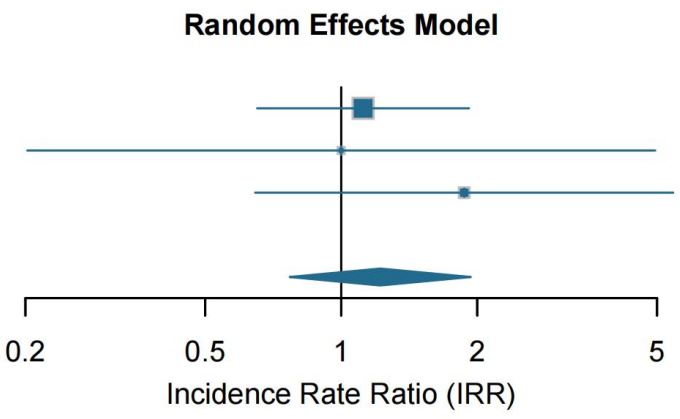

B

Fatigue

Study	Experimental Events	Experimental Person−Time	Control Events	Control Person−Time	IRR	95% CI	Weight (%)
Targan 1997	9	27.24	1	3.31	1.09	[0.14; 8.63]	37.5
Present 1999	6	21.81	2	10.73	1.48	[0.30; 7.31]	62.5
**Common effect model**					**1.32**	**[0.37; 4.67]**	**100.0**

Heterogeneity: I^2 = 0.0%, τ^2 = 0, p = 0.8221

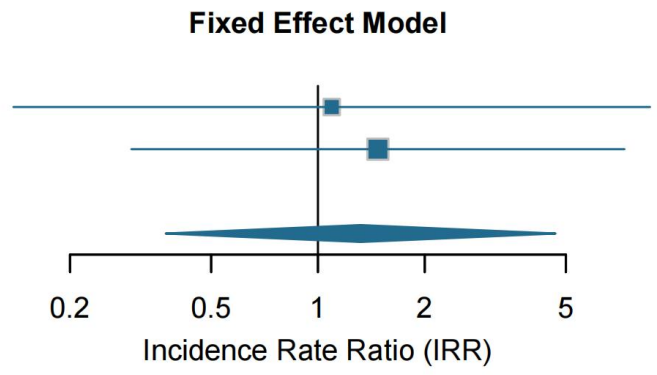

Headache

Study	Experimental Events	Experimental Person−Time	Control Events	Control Person−Time	IRR	95% CI	Weight (%)
Present 1999	11	21.81	7	10.73	0.77	[0.30; 1.99]	39.8
Rutgeerts 1999	6	23.49	4	19.83	1.27	[0.36; 4.49]	22.3
Targan 1997	22	27.24	5	3.31	0.53	[0.20; 1.41]	37.9
**Common effect model**					**0.75**	**[0.41; 1.36]**	**100.0**

Heterogeneity: I^2 = 0.0%, τ^2 = 0, p = 0.5686

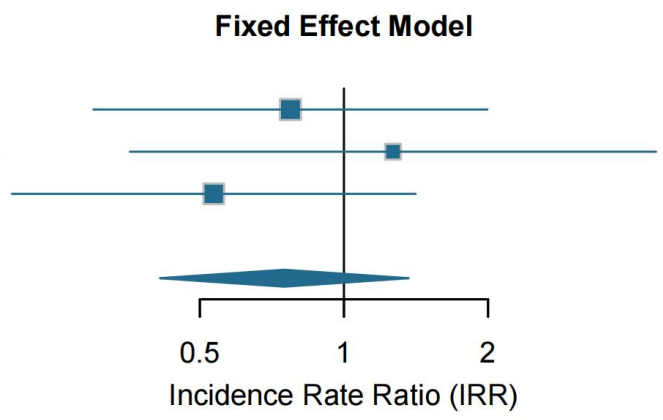

Supplementary Figure 5 A Forest plot illustrating IRR of ustekinumab on fatigue, headache and arthritis/arthralgia vs. placebo in subgroup analyses. CI confidence interval. **B** Forest plot illustrating IRR of infliximab on fatigue and headache vs. placebo in subgroup analyses. CI confidence interval.

Fatigue

Study	Experimental Events	Experimental Person−Time	Control Events	Control Person−Time	Fixed Effect Model	IRR	95% CI	Weight (%)
Regueiro 2016	16	269.31	18	239.78		0.79	[0.40; 1.55]	39.9
Colombel 2010	26	142.20	25	108.10		0.79	[0.46; 1.37]	60.1
**Common effect model**						**0.79**	**[0.52; 1.21]**	**100.0**

Heterogeneity: I^2 = 0.0%, τ^2 = 0, p = 0.9981

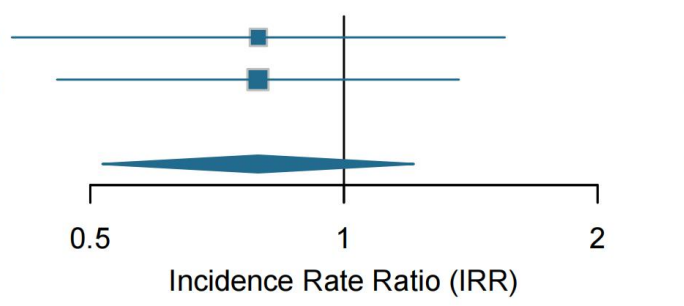

Headache

Study	Experimental Events	Experimental Person−Time	Control Events	Control Person−Time	Fixed Effect Model	IRR	95% CI	Weight (%)
Hanauer 2024	18	200.81	5	88.59		1.59	[0.59; 4.28]	12.1
Regueiro 2016	37	269.31	34	239.78		0.97	[0.61; 1.54]	54.8
Colombel 2010	23	142.20	20	108.10		0.87	[0.48; 1.59]	33.1
**Common effect model**						**0.99**	**[0.70; 1.40]**	**100.0**

Heterogeneity: I^2 = 0.0%, τ^2 = 0, p = 0.5924

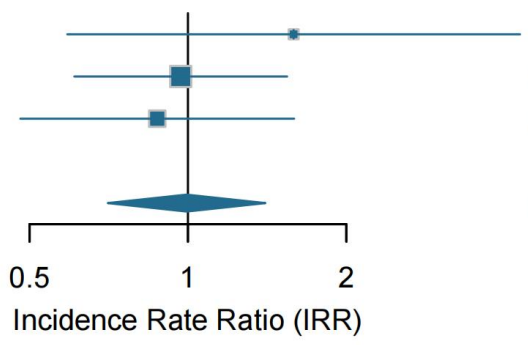

Supplementary Figure 6 Forest plot illustrating IRR of infliximab on fatigue and headache vs. placebo in subgroup analyses. CI confidence interval.

Fatigue

Study	Events	Experimental Person−Time	Events	Control Person−Time	Random Effects Model	IRR	95% CI	Weight (%)
Sandborn 2012	3	64.15	9	21.83		0.11	[0.03; 0.42]	43.7
Sandborn 2022	42	1788.23	8	177.72		0.52	[0.24; 1.11]	56.3
**Random effects model**						**0.27**	**[0.06; 1.18]**	**100.0**

Heterogeneity: I^2 = 74.5%, τ^2 = 0.8677, p = 0.0476

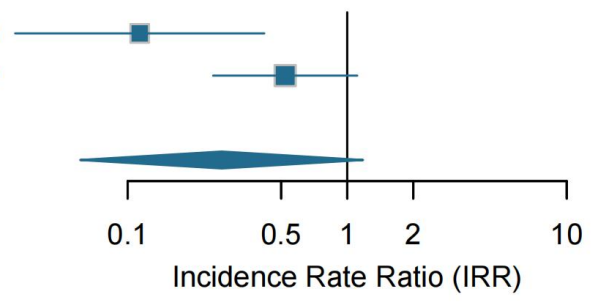

Headache

Study	Events	Experimental Person−Time	Events	Control Person−Time	Fixed Effect Model	IRR	95% CI	Weight (%)
Sandborn 2012	12	87.28	11	87.34		1.09	[0.48; 2.47]	28.7
Sandborn 2022	59	1788.23	12	177.72		0.49	[0.26; 0.91]	49.9
Ferrante 2024	15	293.30	6	119.50		1.02	[0.40; 2.63]	21.4
**Common effect model**						**0.72**	**[0.46; 1.12]**	**100.0**

Heterogeneity: I^2 = 33.5%, τ^2 = 0.0943, p = 0.2222

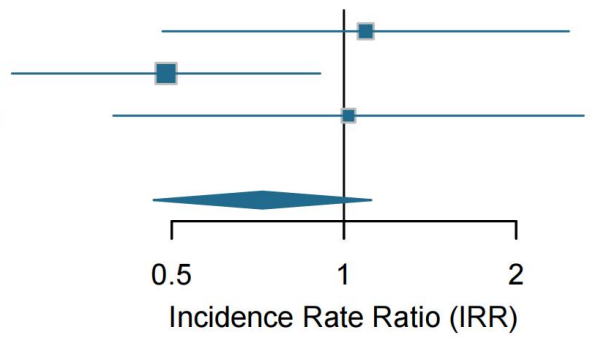

Arthritis/arthralgia

Study	Events	Experimental Person−Time	Events	Control Person−Time	Fixed Effect Model	IRR	95% CI	Weight (%)
Sandborn 2012	11	87.28	15	87.34		0.73	[0.34; 1.60]	30.1
Sandborn 2022	72	1788.23	12	177.72		0.60	[0.32; 1.10]	48.8
Ferrante 2024	8	293.30	10	119.50		0.33	[0.13; 0.83]	21.1
**Common effect model**						**0.56**	**[0.36; 0.86]**	**100.0**

Heterogeneity: I^2 = 0.0%, τ^2 < 0.0001, p = 0.4053

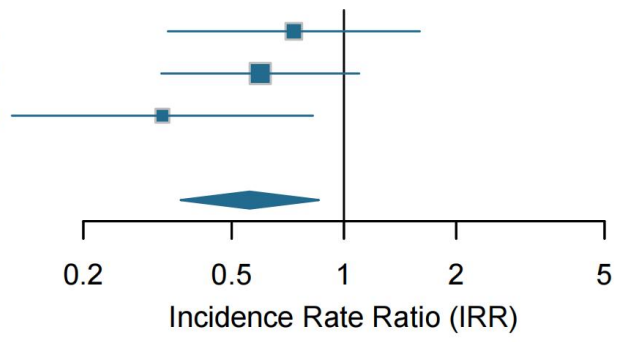

Supplementary Figure 7 Forest plot illustrating IRR of ustekinumab on fatigue, headache and arthritis/arthralgia vs. placebo in subgroup analyses. CI confidence interval.

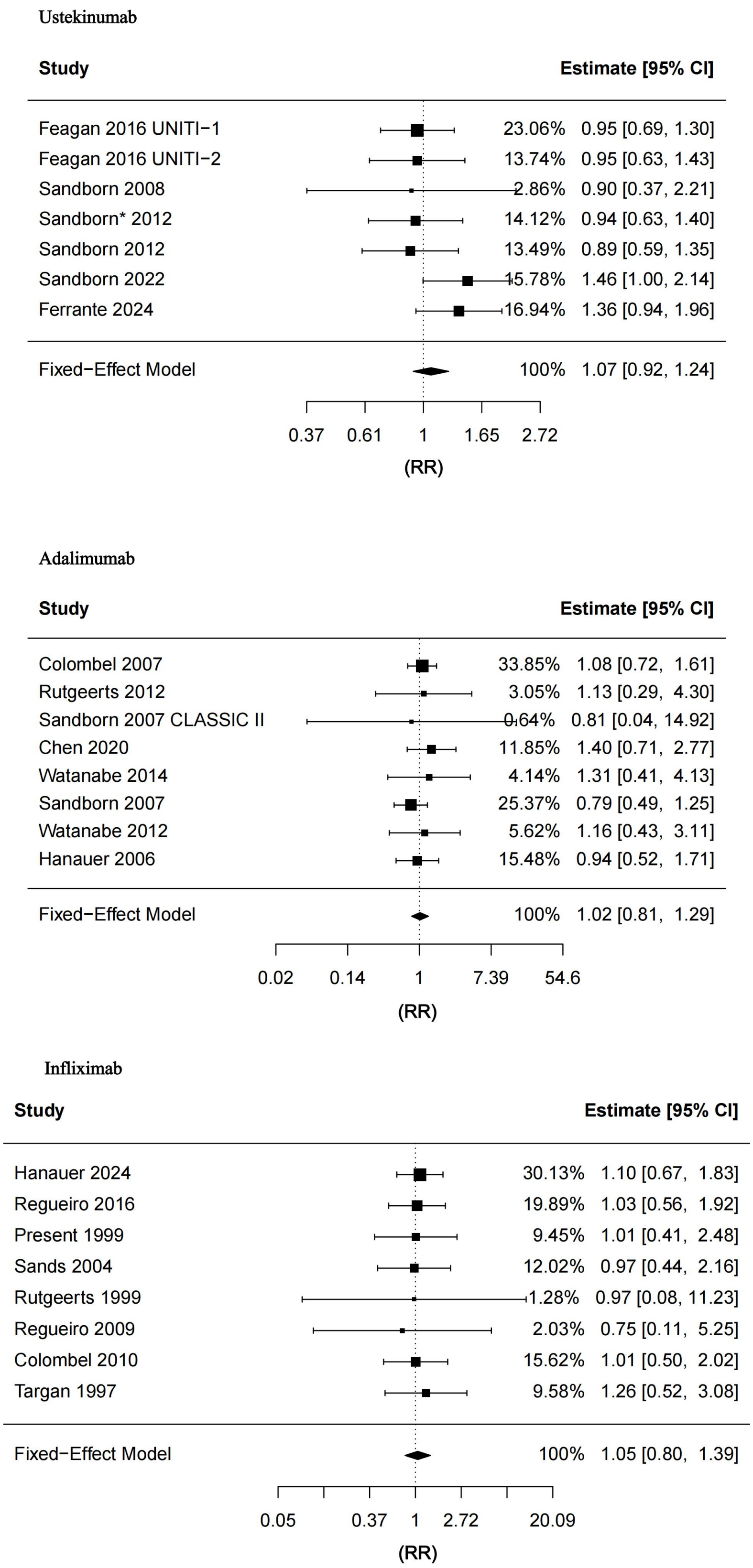

Supplementary Figure 8 Forest plot illustrating RR of ustekinumab, adalimumab and infliximab on adverse event vs. placebo. RR rate ratios; CI confidence interval.

Ustekinumab

Study	Experimental Events	Experimental Person−Time	Control Events	Control Person−Time	IRR	95% CI	Weight (%)
Feagan 2016 UNITI−1	45	79.08	22	39.07	1.01	[0.61; 1.68]	15.0
Feagan 2016 UNITI−2	24	65.69	19	32.61	0.63	[0.34; 1.14]	12.5
Sandborn 2008	5	8.00	3	8.00	1.67	[0.40; 6.97]	3.3
Sandborn* 2012	25	64.15	15	21.83	0.57	[0.30; 1.08]	11.6
Sandborn 2012	40	87.28	47	87.34	0.85	[0.56; 1.30]	18.0
Sandborn 2022	277	1788.23	36	177.72	0.76	[0.54; 1.08]	20.8
Ferrante 2024	48	293.30	48	119.50	0.41	[0.27; 0.61]	18.8
**Random effects model**					**0.70**	**[0.53; 0.92]**	**100.0**

Heterogeneity: $I^2 = 50.1\%$, $\tau^2 = 0.0637$, $p = 0.0615$

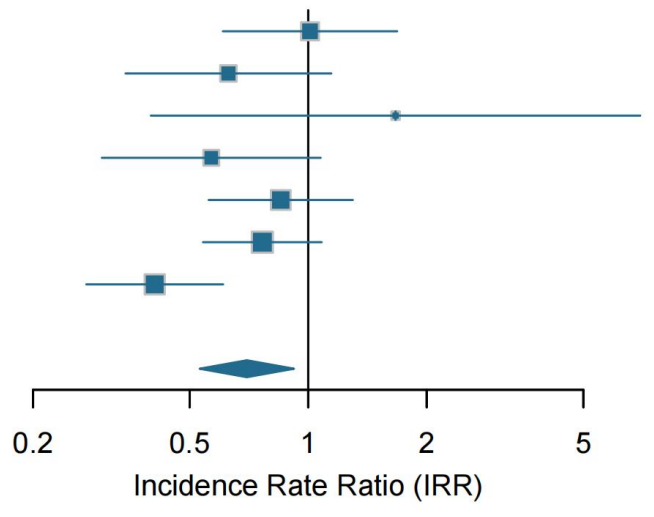

Adalimumab

Study	Experimental Events	Experimental Person−Time	Control Events	Control Person−Time	IRR	95% CI	Weight (%)
Colombel 2007	45	486.19	40	211.29	0.49	[0.32; 0.75]	68.6
Rutgeerts 2012	4	34.20	5	21.40	0.50	[0.13; 1.86]	7.2
Sandborn 2007 CLASSIC II	1	39.85	2	19.39	0.24	[0.02; 2.68]	2.2
Chen 2020	2	7.84	1	7.92	2.02	[0.18; 22.28]	2.2
Watanabe 2014	2	12.00	6	17.00	0.47	[0.10; 2.34]	4.9
Sandborn 2007	2	12.23	8	12.76	0.26	[0.06; 1.23]	5.2
Watanabe 2012	4	5.15	2	1.77	0.69	[0.13; 3.75]	4.3
Hanauer 2006	4	17.30	3	5.69	0.44	[0.10; 1.96]	5.6
**Common effect model**					**0.48**	**[0.34; 0.69]**	**100.0**

Heterogeneity: $I^2 = 0.0\%$, $\tau^2 = 0$, $p = 0.9290$

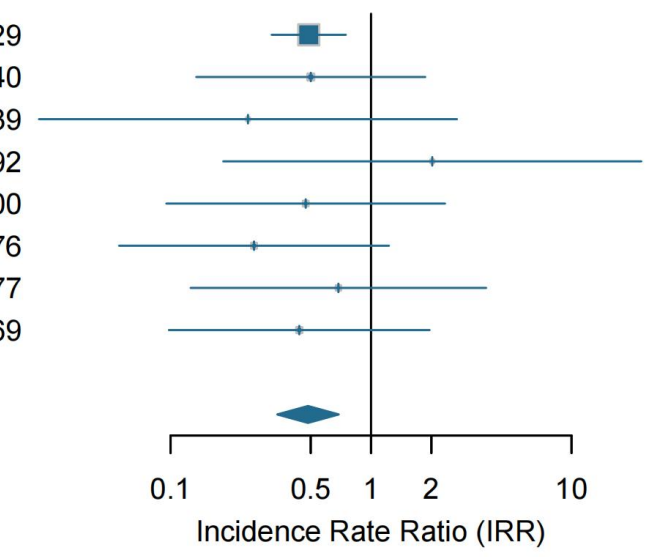

Infliximab

Study	Experimental Events	Experimental Person−Time	Control Events	Control Person−Time	IRR	95% CI	Weight (%)
Hanauer 2024	16	200.81	8	88.59	0.88	[0.38; 2.06]	6.1
Regueiro 2016	39	269.31	36	239.78	0.96	[0.61; 1.52]	21.3
Sands 2004	19	106.92	33	110.00	0.59	[0.34; 1.04]	13.7
Hanauer 2002	97	385.70	55	157.60	0.72	[0.52; 1.00]	40.0
Colombel 2010	27	142.20	43	108.10	0.48	[0.29; 0.77]	18.9
**Common effect model**					**0.70**	**[0.57; 0.86]**	**100.0**

Heterogeneity: $I^2 = 20.1\%$, $\tau^2 = 0.0187$, $p = 0.2866$

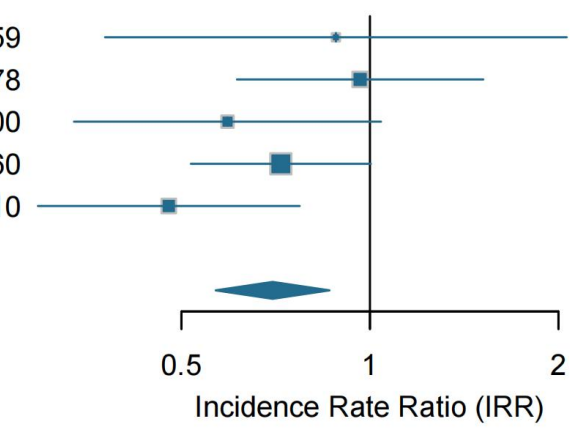

Supplementary Figure 9 Forest plot illustrating IRR of ustekinumab, adalimumab and infliximab on serious adverse event vs. placebo. CI confidence interval.
